# RNA-Sequencing Reveals the Progression of Phage-Host Interactions between φR1-37 and *Yersinia enterocolitica*

**DOI:** 10.3390/v8040111

**Published:** 2016-04-22

**Authors:** Katarzyna Leskinen, Bob G. Blasdel, Rob Lavigne, Mikael Skurnik

**Affiliations:** 1 Department of Bacteriology and Immunology, Medicum, and Research Programs Unit, Immunobiology, University of Helsinki, P.O.Box 21 (Haartmaninkatu 3), FIN-00014 HY Helsinki, Finland; katarzyna.leskinen@helsinki.fi; 2 Laboratory of Gene Technology, KU Leuven, BE-3001 Leuven, Belgium; blasdelb@gmail.com (B.G.B.); rob.lavigne@biw.kuleuven.be (R.L.); 3 Division of Clinical Microbiology, Helsinki University Hospital, HUSLAB, FIN-00270 Helsinki, Finland

**Keywords:** *Yersinia enterocolitica*, bacteriophage, φR1-37, transcriptome

## Abstract

Despite the expanding interest in bacterial viruses (bacteriophages), insights into the intracellular development of bacteriophage and its impact on bacterial physiology are still scarce. Here we investigate during lytic infection the whole-genome transcription of the giant phage vB_YecM_φR1-37 (φR1-37) and its host, the gastroenteritis causing bacterium *Yersinia enterocolitica*. RNA sequencing reveals that the gene expression of φR1-37 does not follow a pattern typical observed in other lytic bacteriophages, as only selected genes could be classified as typically early, middle or late genes. The majority of the genes appear to be expressed constitutively throughout infection. Additionally, our study demonstrates that transcription occurs mainly from the positive strand, while the negative strand encodes only genes with low to medium expression levels. Interestingly, we also detected the presence of antisense RNA species, as well as one non-coding intragenic RNA species. Gene expression in the phage-infected cell is characterized by the broad replacement of host transcripts with phage transcripts. However, the host response in the late phase of infection was also characterized by up-regulation of several specific bacterial gene products known to be involved in stress response and membrane stability, including the Cpx pathway regulators, ATP-binding cassette (ABC) transporters, phage- and cold-shock proteins.

## 1. Introduction

Like all viruses, bacteriophages rely heavily on the host metabolism and must co-opt host processes to complete a productive infection. Therefore, the course of phage infection is a complex struggle between the virus and the bacterial host [1]. The lytic infection process involves a number of programmed steps: adsorption to the susceptible bacterial cell, injection of the viral genome, transition from host to a phage-directed metabolism, replication of the viral genome, morphogenesis, packaging of the viral particles and finally the lysis of the host cell [2]. Current knowledge of phage-host interactions, however, is based largely on a small number of *Escherichia coli* phages, whereas the insight of the course infection of phages in different hosts remains limited [3,4].

While numerous phages infecting the members of *Yersinia* genus have been identified [5,6,7,8], only a few have been characterized in detail. Recent interest in bacteriophages has been spurred by the prospect that knowledge of phage-host interactions, as well as the small and early phage proteins that drives them, can result in development of new antibacterial agents. Indeed, properly characterized, active bacteriophage can be used directly in phage therapy (reviewed in [9,10,11]). Additionally, among the bacteriophage genes, new potential antimicrobial agents can be identified (reviewed in [12,13,14]).

Bacteriophage φR1-37 was isolated from sewage, and selected for its ability to infect strain YeO3-R1, a spontaneous mutant of *Yersinia enterocolitica* serotype O:3 lacking the O-antigen from its lipopolysaccharide (LPS) [15]. The host range of φR1-37, as well as genetic and structural data, revealed that the LPS outer core (OC) hexasaccharide serves as the receptor for the phage [15,16,17,18]. Later, it was shown that φR1-37 can recognize several surface receptors, which broadens its host range and allows the phage to infect different serotypes of *Y. enterocolitica*, including O:3, O:5,27, O:9 and O:50, as well as *Y. similis* O:9 [19,20]. φR1-37 has a lytic life cycle with eclipse and latent periods of 40 and 50 min, respectively. The burst size is estimated to be about 80 plaque forming units (PFU) per infected cell [16]. The structural analysis of the φR1-37 revealed that it is a large tailed virus belonging to the *Myoviridae* family of viruses. Phage particles have an icosahedral head of 138 nm in diameter, a short neck of 15 nm, a long contractile tail of 383 nm and tail fibers of about 75 nm. The genome is 262,391 bp in size and comprises of 367 protein coding and 5 tRNA coding genes. Of the encoded proteins, only 140 proteins have a putative function either by similarity to known proteins or by the fact that they were detected as structural virion proteins [21]. Furthermore, the φR1-37 genome is composed of DNA in which thymidine is completely replaced by deoxyuridine. However, the few phage encoded gene products showing similarity to proteins predicted to participate in nucleotide metabolism did not explain the strategy used by φR1-37 to redirect host nucleotide metabolism from the usage of thymidine to deoxyuridine [21]. φR1-37 shares several similarities, like the genome size, capsomer arrangements and the type I and II surface protrusions, with the *Pseudomonas aeruginosa* bacteriophage φKZ [16,21,22]. The study of the transcriptional scheme of φKZ revealed the presence of early, middle and late genes that are solely transcribed by the phage-encoded RNA polymerases (RNAP). Moreover, the transcriptomic study showed the time-dependent accumulation of the φKZ transcripts, but remarkably only one bacterial operon was significantly up-regulated during the infection [3].

In this study, we used RNA sequencing to elucidate the gene expression pattern of φR1-37 and to assess the global effect of the phage on the host transcription in a single infection event. The phage and host transcriptomes obtained at different time points during the lytic infection cycle allowed us to gain insight of the gene expression patterns in the course of infection, of the transcriptional overtake and the bacterial responses provoked by φR1-37 phage in its *Y. enterocolitica* host.

## 2. Materials and Methods

### 2.1. Bacterial Strains, Phage Propagation, and Growth Conditions

Bacteriophage φR1-37 was propagated in *Y. enterocolitica* O:3 strain YeO3-R1 [15] as described previously [16]. *Y. enterocolitica* was grown in lysogeny broth (LB) at room temperature. Luria agar (LA) plates were used for all solid cultures and prepared by supplementing LB with 1.5% Bacto agar.

### 2.2. Growth Curves

Overnight (16 h) bacterial cultures were diluted 1:10 in fresh LB medium and 180 µL aliquots were distributed into honeycomb plate wells (Growth Curves Ab Ltd., Helsinki, Finland) where they were mixed with 20 µL aliquots of 10^−2^–10^−6^ dilutions of φR1-37 phage stock (1 × 10^8^ PFU/mL). The approximate multiplicity of infection (MOI) thus ranged from 10^−1^ to 10^−5^. In the positive control cultures the phage was replaced with 20 µL of medium, and the negative controls were prepared by mixing 20 µL of phage stock with 180 µL of medium. The growth experiments were carried out at 4 °C, 16 °C, 22 °C, and 37 °C using the Bioscreen C incubator (Growth Curves Ab Ltd.) with continuous shaking. The optical density (OD) OD_600_ values were measured at selected time intervals. The averages were calculated from values obtained for the bacteria grown in eight parallel wells.

### 2.3. Total RNA Extraction

For the transcriptome analyses, YeO3-R1 bacteria were grown for 16 h at room temperature (22 °C) and subsequently diluted 1:10 in fresh LB to a total volume of 10 mL. The OD_600_ of the culture was measured during the incubation period and, when it reached 0.6, the culture was divided into two identical parts. One part was infected with phage φR1-37 at MOI of 10, the other served as uninfected control. Next, both cultures samples were incubated under identical conditions and the OD_600_ of the cultures were measured to ensure proper course of infection. Samples for RNA isolation were taken (1 mL) from the non-infected culture (negative control) and from the infected culture at various time points post-infection (2, 5, 10, 15, 21, 28, 35, 42, 49 min). Total RNA was isolated from the samples using the SV Total RNA Isolation System (Promega, Madison, Wisconsin, US). The quality and the ribosomal RNA (rRNA) profiles of the isolated RNA preparates were analyzed using Bioanalyzer (Agilent, Santa Clara, California, US) and the RNA 6000 Nano Kit (Agilent).

### 2.4. RNA Sequencing

The RNA-sequencing and data analysis were performed at the Institute for Molecular Medicine Finland (FIMM) Technology Center Sequencing Unit. The rRNA was removed using Ribo-Zero^TM^ rRNA Removal Kit for Gram-negative Bacteria (Epicentre, Madison, Wisconsin, US). Paired-end sequencing was performed on Illumina HISeq2000 sequencer (Illumina, San Diego, California, US) with the read length of 90 nucleotides. The RNA sequence data has been deposited to Gene Expression Omnibus (Acc. no GSE77068).

### 2.5. Computational and Statistical Analyses

Raw sequencing reads were trimmed and filtered for quality before being mapped to the host and phage genomes using the CLC genomics workbench v7.5.1. The Total Gene Reads (TGR) aligning to each non-rRNA gene feature in both the phage and host genomes were counted.

Genes were considered as differentially expressed if the calculated log_2_ value of the fold change (log2FC) value of TGR was over 1.5 or below −1.5, and the negative control value was less than sample mean count value minus 2 standard deviations or higher than sample mean count value plus 2 standard deviations. In the comparison performed between early and late bacterial response differential expression was tested using the Student *t*-test. The differential expression was considered as statistically significant if *p* < 0.01. Those comparisons that include the 0 min negative control are indeed susceptible to significant culture bias as there is only a single replicate for that condition.

After normalization by Total Count [23], clustering of the φR1-37 temporal patterns was performed as follows. The averages of 2–5 min (early), 10–21 min (middle), 28–49 min (late) TGR values were calculated and presented as percentage values with the highest value set as 100%. Genes were classified according to which phase the highest average value corresponded to. If the difference of average values between different phases was less than 40% the genes were considered as not regulated. Genes with the average TGR < 10 were not considered further.

## 3. Results and Discussion

### 3.1. Microbiological Parameters of the φR1-37 Infection Process

**Figure 1:**
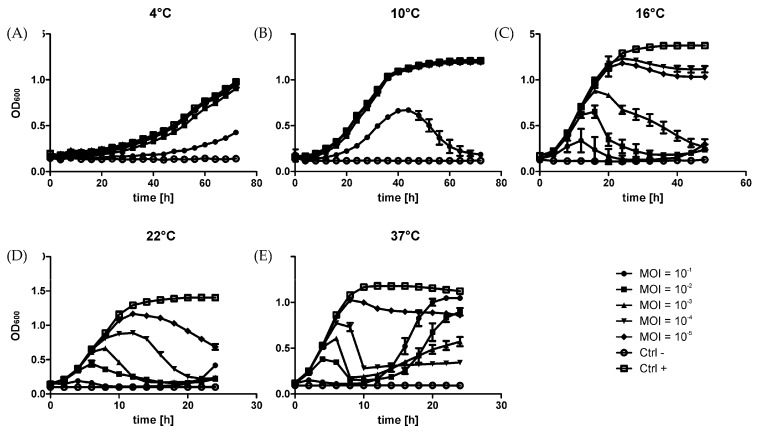
Growth curves of YeO3-R1 bacteria at different temperatures when infected with phage φR1-37. Bacteria infected with phage φR1-37 at MOIs of 10^−1^ to 10^−5^ were grown in LB at 4 °C (**A**), 10 °C (**B**), 16 °C (**C**), 22 °C (**D**), and 37 °C (**E**). Each data point in the graphs represents the average of eight replicates. The error bars represent the standard deviation for the optical density calculated for each time point. Note the different axis scales in the different panels.

The infection dynamics of phage φR1-37 infection has been previously defined using the one-step growth curve method showing that the eclipse period lasts 40 min, and the latent period 50 min [16]. Here, we further analyzed the phage-host interplay under different temperature conditions using Bioscreen C. The results show that φR1-37 establishes infection in YeO3-R1 at all the studied temperatures, though its effectiveness increases with temperature (Figure 1). Both at four and 10 °C, only the highest number of phage (*i.e.*, MOI = 100) was able to influence the bacterial growth. At 10 °C, the cultures were followed long enough to see the lysis of the bacteria that took place slowly between 40 and 70 h of incubation (Figure 1). At higher temperatures, the infections proceeded also with all the other used MOI values, the bacterial growth curves nicely reflecting the range of MOIs. At 16, 22 and 37 °C, regrowth of bacteria could be detected after 40, 20 and 15 h, respectively, in the (fully) lysed cultures. This experiment demonstrates that phage φR1-37 is capable of establishing efficient infection over a broad temperature range. The regrowth that followed the complete lysis of the culture at higher temperatures was most likely due to the appearance of phage-resistant bacteria. As the receptor of φR1-37 is the OC of the LPS, phage resistant spontaneous mutants arise with high frequency [16]. Based on these results, the samples for RNA seq analysis were collected from bacteria incubated at room temperature and infected at a MOI = 10.

### 3.2. General Features of the Transcription Analysis

To assess the quality and integrity of total RNA isolated from YeO3-R1 bacteria infected with φR1-37 phage a Bioanalyzer (Agilent) run was performed. The results indicated that the RNA quality was sufficient for library preparation and RNA-sequencing (Figure S1). The sample quality was inspected visually, as the RNA integrity number (RIN) could not be counted due to the presumed splicing of the 23S rRNA in *Y. enterocolitica* O:3 [24]. However, the Bioanalyzer electrophoresis gel revealed significant degradation of RNA samples that increased progressively over the course of infection (Figure S2). Coliphage T4 terminates the expression of host genes, not only by affecting their transcription, but also by altering the stability of existing mRNAs [25]. While it is unclear what is responsible for the observed degradation, or whether it extends past rRNA, it is possible that this is a result of φR1-37 using similar mechanisms to overtake the host cell metabolism and thereby changes the stability of different host RNA species leading to their partial degradation.

**Figure 2:**
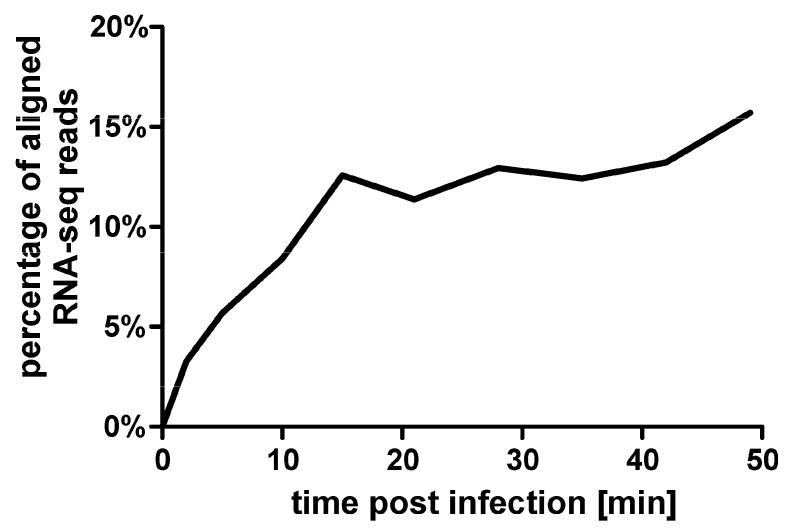
Percentage of RNA sequencing reads aligning to the φR1-37 phage genome at different time points post-infection.

To analyze changes in the transcriptome, the RNA sequencing reads were aligned to both the host and phage genomes in a strand-specific manner. Our results indicate a progressively increasing replacement of host reads with phage reads, starting from 3.27% in the 2 min sample and peaking at 15.71% in the last time point (Figure 2; Table S1). This represents a relative accumulation of phage transcripts that is significantly lower compared to the 45% found in φKZ infection [3].

A principal component analysis (PCA) assessing the co-variance between each sample showed progressive differentiation between the samples of following time points and the distinctive difference between the non-infected negative control and the first time points (Figure S3). Based on these results, the single time point samples could be joined into four groups to allow a relevant statistical analysis: early phase (2 and 5 min), middle phase (10, 15, and 21 min), late phase (28, 35, 42, and 49 min), and non-infected negative control taken immediately before infection (0 min). Importantly, these groups represent technical replicates taken from the same infection flask.

### 3.3. Temporal Regulation of φR1-37 Transcription

**Figure 3:**
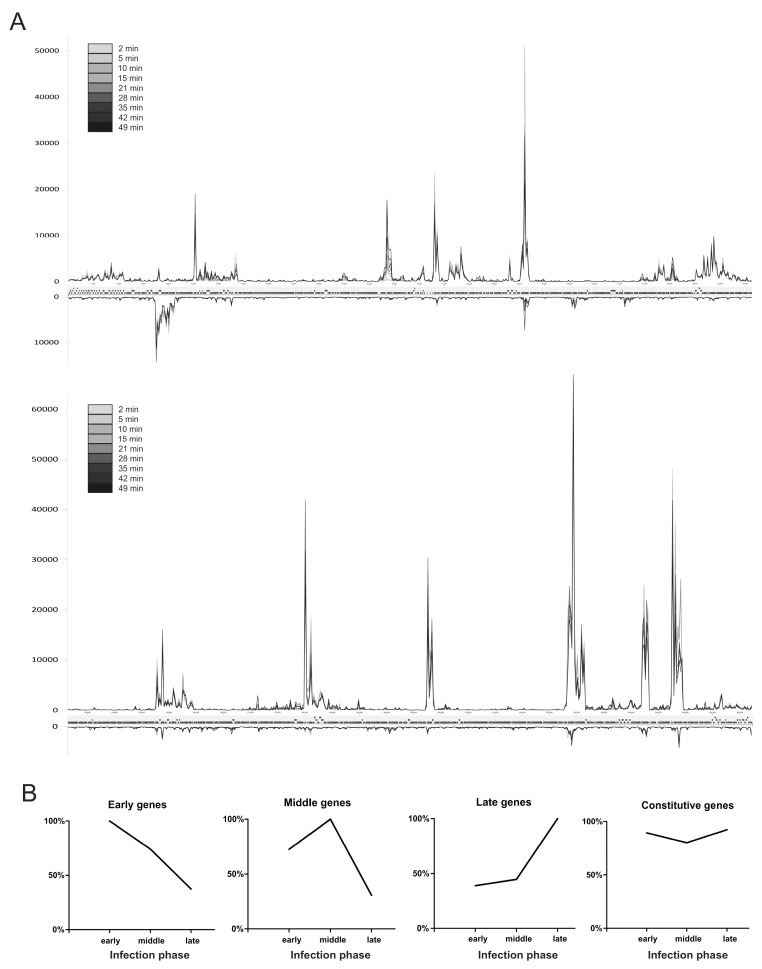
The progression of φR1-37 genome transcription during the infection cycle. The number of reads aligning to every 250 bp fragment of both strands of the phage genome was plotted for each time point (**A**). The intensity of the color of the curves from grey to black indicate the consecutive time points (for high resolution image of Panel A, see Figure S4). Different temporal classes of φR1-37 gene expression (**B**). For each gene and infection phase the highest Total Gene Reads (TGR) value was set to 100% and the other values set accordingly. The genes were grouped into four temporal classes and the curves represent the averages calculated for these.

Phage transcription at each measured point in infection can be visualized by summarizing the reads aligning to either strand of the phage genome for every 250 bp into a count table, normalizing the reads aligning to the phage in each sample against each other by Total Count (TC), and plotting each strand separately (Figure 3A). This profile shows a generally stable gene transcription pattern throughout the early, middle and late phases. Most of the genes analyzed present fairly similar pattern of expression in all the studied time points. Only selected genes showed clear differences during expression when comparing the early and late phase of infection (Table S2). The comparison of average read counts for early (2–5 min), middle (10–21 min) and late (28–49 min) phases for individual genes indicated the existence of four distinctive patterns of gene expression (Figure 3B). The first group comprises of 92 φR1-37 genes, whose transcripts accumulated during the first 10 min of infection and could therefore classified as early genes. Transcript abundance of the second group (8 genes) reached the maximal level between 15 and 28 min post-infection, allowing classifying as middle genes. The number of transcripts of the third group classifying 94 genes as late, increased over time, reaching its peak between 35 and 49 min. Finally, the largest fourth group comprised 147 φR1-37 genes that presented no significant changes in their abundance at different time points post-infection and they were classified as constitutive genes. Out of 367 genes, 26 were excluded due to their very low expression levels under these sampling conditions. No clear associations between the genomic locations of the genes and the expression patterns were observed. These findings do deviate completely from the typical temporal pattern with a shift in transcripts from early to late genes and clustering of the same class genes in the genome, as observed for several other phages using either RNA-seq [3,4] or older methods [26,27,28,29]. As most φR1-37 genes lack clear functional annotations, it is at present time not even possible to speculate on many of the constitutively expressed genes.

Of the 92 early genes, 87 showed no similarity to known genes (Table S2). The other early genes encoded an endo-type membrane-bound lytic murein transglycosylase (g331), a Pro-Ala-Ala-Arg (PAAR)-repeat containing protein (g142) and, unexpectedly, two virion structural proteins (g088, g094). Only one of the 18 middle genes had a predicted function, a putative RNA-binding protein (g284). Most of the genes involved in the DNA replication and repair (22 out of 30) were classified as late genes. The late genes (*n* = 94) contained a.o. 20 genes encoding virion structural proteins, four nucleotide metabolism genes and three RNAP and RNA interaction genes. Interestingly, most of the genes coding for virion structural proteins (48 out of 70) were classified as constitutive, showing a stable expression throughout the infection. Similarly, seven genes coding for DNA replication and repair factors, two genes coding for ATPases, and three genes coding for factors involved in nucleotide metabolism were classified as constitutive (Table S2). Additionally, two genes annotated to encode lysis-related proteins (g084 and g289) also belonged to the same class although their expression level was very low compared to the g331 encoding lysis protein that was one of the genes with the highest TGR values (Table S2). Further studies are warranted to establish experimentally the function of g084 and g289 products and the possible impact of their constitutive expression on the lysis of the host. While our observation that the two genes predicted to be involved in lysis runs counter to the standard infection model [30] it may indicate a complex mechanism of lysis inhibition like is found in phage T4 [31].

On the other hand, among the genes having the highest TGR values we identified genes coding for virion structural proteins (g326, g176, g083, g174, g099), ATPases (g304, g061) and for the lysis protein (g331). No reads aligned to genes g067, g106, g109, g116, g165, g166, g172, g180, g181, g188, g200, g201, g217, g232, g288, g312, g315, g316, and g357. Detailed information about the phage gene expression is presented in the Table S2.

The RNA sequencing data supports our previous Northern blot results, presenting the general snapshot of the φR1-37 transcription [21]. Consistent with Northern blotting we here show that the genes g072-g072, g103, g170 and g329 are expressed late and that the g231 gene expression is constitutive. The discrepant results for genes g048-g049, g145, g281 and g298 can be explained by the very low TGR values of their transcripts. The low abundance of these transcripts and the lower abundance of phage transcripts in general, during the early time points, makes the Northern blot analysis challenging. In this situation, the highly sensitive RNA sequencing proves to be a more reliable method.

Earlier studies showed that the genes encoding proteins with similarities to bacterial RNAP β and β′ subunits are conserved in all known φKZ-related phages [32]. In φKZ, four genes encoding constitutive viral RNAP are middle and/or late genes, whereas the genes coding for nonvirion RNAP are expressed in the early phase [3]. *In vitro*, the RNAP complex of φKZ initiates transcription from late phage promoters in a rifampicin resistant manner. Moreover, φKZ RNAP does not possess assembly and promoter specificity subunits characteristic for bacterial, archaeal or eukaryote polymerases [32]. Our study shows that in φR1-37, the g274, g102 and g261 genes coding for RNAP subunits are late and highly expressed. The remaining three (g178, g231 and g099), are expressed constitutively from the earliest points of infection and they are characterized by medium to high transcript abundance. It was previously assumed that φR1-37 introduces β and β′ subunits to the host cell to take over the transcription early after infection [21,33]. It is possible, that similar to φKZ, the φR1-37 phage depends strongly on its own RNAP and uses it throughout the entire course of infection.

### 3.4. Validation of Phage Regulatory Elements

The previously performed computational search for regulatory elements (PHIRE/MEME [34,35]) predicted a number of putative φR1-37 promoters characterized by a A-dU rich consensus sequence [21]. The RNA sequencing experimentally confirmed 56 of the predicted, conserved promoters, and supports the previously published consensus sequence to a large extent (Figure S5). No new conserved sequences were observed in regions upstream of putative new transcription starting sites. Skurnik *et al.* (2012) also predicted 16 putative sigma-70 promoters. However, the RNA-seq data analysis revealed that these are mainly weak and middle strength promoters, located upstream of the genes encoding virion structural proteins. The transcription starting from these promoters is stable during the course of infection and leads to production of transcripts of low to middle abundance. Distinct expression patterns resulting from factors other than differences in basal promoter elements may explain the absence of differentiation between the early and late promoters at the sequence level. These promoters could potentially be regulated by the appearance of transcription factors, by modifications of RNAP or by differences in intrinsic promoter strengths [26].

### 3.5. Novel RNA Species in φR1-37 Genome

This work reveals the presence of 10 previously unannotated RNA species being expressed from the φR1-37 genome (Table 1). Nine of them are encoded from the non-sense strand thus being putative antisense RNAs (asRNAs). The remaining small RNA was encoded in an intragenic region with no open reading frame possibilities. However, the data did not confirm the previously postulated antisense RNA species antisense to g233 [21,36].

The presence of small non-coding RNAs (ncRNAs) in bacteriophage genomes has been established, yet their role is not understood. Presumably, the identified asRNA species function as a supplementary component of the gene expression regulation system affecting the phage mRNA transcribed from the sense strand. Small asRNAs might play a role in regulation of expression of complementary mRNA by interfering with sense RNA transcription [37]. Moreover, asRNA species may protect the primary transcript by covering the single-stranded binding sites of endoribonuclease E, or they may inhibit the translation of mRNA by blocking the ribosome binding site [38,39].

It is also possible, that the intragenically encoded misc_4 small RNA (sRNA) species targets the host mRNA. The analysis performed with TargetRNA2 [40] with the threshold set up to *p* < 0.001 resulted in three host genes: *ptr* encoding the protease III, *tufA* encoding elongation factor Tu and *ddrA* encoding the propanediol utilization diol dehydratase reactivation protein. However, none of these genes showed differential expression in our study. Still, it remains possible that the sRNA has a post-transcriptional effect and regulates the production of the final product affecting the translation of the mRNA.

### 3.6. Transcriptional Response of YeO3-R1 to φR1-37 Infection

To elucidate the host response to the φR1-37 infection, we compared the bacterial transcriptome before infection (negative control) to the early (2–10 min) and late time points (28–49 min) post-infection (Tables S3 and S4). Moreover, to get a broader view on the host response changes during the course of infection, we compared the bacterial transcriptome from the early to the late phase of infection (Table S5). The early bacterial response indicates that the transcript levels of approximately 2.58% (112 genes out of 4349 genes in total) of the *Y. enterocolitica* genes decreases immediately after the phage infection. Only a very small fraction of the differentially expressed genes (seven genes), were up-regulated. The up-regulation affected some membrane proteins involved in energy and proton transport, including Na^+^/H^+^ antiporter NhaA (Y11_38431; Log2FC = +3.35), glycerol dehydrogenase (Y11_37711; +3.09), transcriptional activator NhaR (Y11_38441; +2.10), Mg^2+^ transport protein C (Y11_14731; +1.75) and ATP synthase protein I (Y11_29681; +1.25). Among the large number of down-regulated genes, the most prominently affected include the transcriptional regulator of the GntR family (Y11_21251; −3.81), nitrite reductase large subunit (Y11_32431; −3.24), ADP-glucose synthase (Y11_14901; −2.80), as well as several membrane proteins. This type of bacterial response is not distinct from other lytic host-phage systems, where a general decrease in the expression of host genes occurs [41,42].

**Figure 4:**
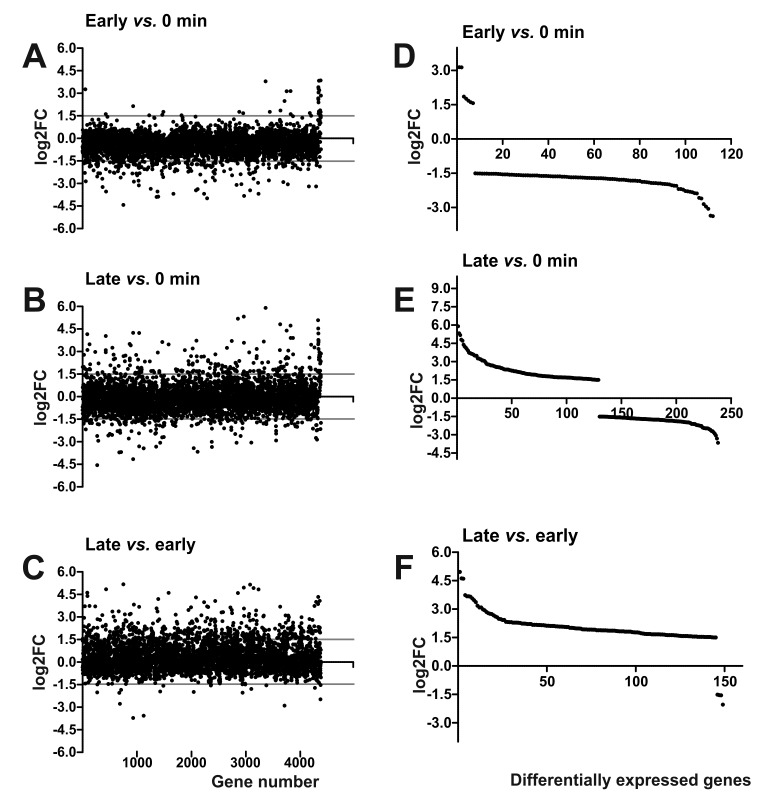
The change in the expression of the bacterial genes during the phage φR1-37 infection cycle. The early phase values represent the average data calculated for the 2 and 5 min time points, and the late phase values, the average for the 28, 35, 42 and 49 min time points. Shown are the Log2FC values between the early phase and the negative control (**A**), between the late phase and negative control (**B**), and between the late and early phases (**C**). Each dot represents the Log2FC value calculated for each gene separately. In the A, B and C graphs, the dots are ordered according to their gene location in the YeO3-R1 genome (the consecutive gene numbers on the X-axis are indicated with the scale at the bottom). The grey lines indicate the selected differential expression threshold of ±1.5. Panels D, E and F demonstrate that in the early phase most differentially expressed host genes are repressed (**D**), while in the late phase many differentially expressed host genes are activated (**E**) and this is even more pronounced when the late phase genes are compared to early phase genes (**F**). For panels D, E and F, the genes are arranged along the X-axis according to their decreasing log2FC values.

The comparison of bacterial transcriptomes of late time points with the pre-infection pattern shows even more pronounced changes in the expression of membrane protein encoding genes (Figure 4). Interestingly, during the late bacterial response, an increase in the number of overexpressed genes was observed: 129 (54.2%) out of 238 differentially expressed genes (5.47% of total *Y. enterocolitica* genes) were overexpressed. Among the most up-regulated genes a phosphate ABC transporter PstS (Y11_29791; +5.35) and Cpx system periplasmic protein (Y11_28711; +5.26) could be identified. Moreover, the bacterial response was characterized by up-regulation of several membrane proteins, transporters and stress related genes (including phage-, cold- and osmotic shock proteins). The significance of these changes is not clear at present but it may be either a host or a phage driven response to osmotic stress caused by the rapidly shifting metabolic environment found in some phage infections [43]. Additionally, expression of five transcriptional regulators was changed, three of which (NhaR, PhoB and Ars) were up-regulated and two (YciT and GntR) down-regulated (Table 2).

The comparison of early and late bacterial response shows that several genes, including the Na^+^/H^+^ antiporter NhaA, glycerol dehydrogene and numerous underexpressed genes, were either upregulated or down-regulated from the early time points compared to the final moments of phage infection. However, a significant number (*n* = 149) of genes changed their expression pattern during the course of phage infection (Table S3). Interestingly, the vast majority (145 out of 149) underwent (relative) up-regulation.

Such patterns of overexpression of bacterial genes occurring upon the bacteriophage infection, although unusual to phage-host systems, have been observed previously [4,41]. Similar to the previous cases, we observed overexpression of genes encoding membrane proteins, as well as those involved in transport of small molecules and different types of stress responses. It has been hypothesized previously that bacteriophages may have evolved to make use of the products of some overexpressed host genes [41]. For example, T4 and T7 phages modify host RNase E to carry out the degradation of host mRNA [25]. Our results indicated the up-regulation of the ribonuclease activity A regulator (Y11_28241), which in *E. coli* modulates RNA abundance by binding to RNase E and regulating its activity [44]. The exploitation of stress response genes by the bacteriophages has also been implicated [41]. While our results cannot on their own indicate whether the up-regulation of these genes can be seen as the bacterium attempting to respond to the infection, or the bacteriophage attempting to exploit the responses, they can be regarded as part of the “arms race” between the phage and the bacterium. As weapons in the arms race, the host bacteria have the Clustered regularly-interspaced short palindromic repeats/Cas (CRISPR/Cas) and restriction-modification (RM) systems. While *Y. enterocolitica* completely lacks the CRISPR/Cas system, in theory it could use the restriction enzymes to combat the phage. The restriction enzyme, however, should be present waiting for the phage infection, and if these would threaten the phage it should produce an inhibitor against the restriction enzyme. Alternatively, the phage could shut-off the RM genes. Unfortunately, the RM systems of *Y. enterocolitica* O:3 are more or less completely unknown and very poorly studied. In the genomic annotation two genes putative restriction enzyme encoding genes are present, *Y11_23931* and *Y11_37531*, however, expression of neither of these were significantly affected by the phage infection.

Among the differentially expressed proteins, we identified only two that are involved in nucleotide metabolism (Figure S6). The first one, dGTPase (*Y11_39741*), performs the hydrolysis of dGTP to deoxyguanosine and triphosphate [45]. The predicted aminoacrylate hydrolase (*Y11_13711*) would be required to remove toxic intermediate product in the pyrimidine nitrogen degradation [46]. However, the down-regulated transcription of these two genes after the φR1-37 infection cannot explain the strategy used to redirect the metabolism from the usage of thymidine to deoxyuridine required by the bacteriophage. The expression of other bacterial genes involved in the nucleotide metabolism did not present any significant differences when compared to the negative control. Further studies are warranted to address this question.

## 4. Conclusions

φR1-37 is a giant bacteriophage belonging to the *Myoviridae* family and shows structural similarity to *P. aeruginosa* phage φKZ. Phage φR1-37 recognizes several surface receptors, and thus is capable of infecting different serotypes of *Y. enterocolitica* and *Y. similis* [19,20]. Here, we demonstrate the transcriptional scheme of φR1-37 and describe the impact of φR1-37 infection on the *Y. enterocolitica* YeO3-R1 host strain showing the probable mix of host response and phage meddling. Using the RNA-Seq data we are able to provide a detailed transcriptional narrative for φR1-37 describing the progression of phage infection generally as well as for the shape and temporal context of each phage gene feature specifically. Our study demonstrates that the expression of φR1-37 genes does not follow the standard lytic phage pattern and only selected genes can be classified as typically early, middle or late genes, whereas majority of the genes are being expressed constitutively throughout the course of infection. Moreover, φR1-37 lacks a clear division between the location of the gene in the genome and the observed expression pattern. This feature is not typical for the temporal pattern of gene expression, where a shift in transcripts from early to late genes and clustering of the class genes in the genome occur [4,27,28,29,33].

The analysis of the host transcriptome demonstrated that over 2.5% of the *Y. enterocolitica* genes decreases immediately after the phage infection. Remarkably, the late bacterial response involved an upregulation of over 54% of differentially expressed genes, including the ABC transporters, Cpx system, phage-, cold- and osmotic shock genes. While a decrease in the transcription of host genes has been commonly observed [41,42], the relative increase in abundance of several bacterial transcripts is a rather unique feature.

Global RNA profiling of φR1-37 revealed the presence of ten novel ncRNA species, including one sRNA encoded in the intragenic region. The presence of antisense transcripts has been described previously for other bacteriophages including phage λ and φKZ [3,47,48]. Furthermore, based on the bioinformatics analysis it is assumed that the intragenic sRNA can be targeting a host mRNA, likely being an element of phage-host interaction strategy. Hitherto, the presence of phage sRNA and host RNA interactions were described mainly for ncRNA encoded by the prophages (reviewed in [49]).

In conclusion, we present an extensive description of the global phage-host transcriptome interaction that grounds the further research aiming at elucidation of indicated interactions between these two organisms. Despite the previously observed similarities to *Pseudomonas* phage φKZ, our research suggests that these two bacteriophages differ in regards to gene expression and regulation. The genome of φR1-37 is fairly unique among phages and possesses great number of genes that show no similarity to previously characterized proteins. Generally, in-depth analysis of the mechanism of host gene expression shutoff performed by the phage, as well as the knowledge of the reciprocal interaction between these organisms is crucial for research on novel antibacterial compounds and the development of phage therapy.

## Figures and Tables

**Table 1:** Identified novel non-coding RNA species.

Name	Location in the Genome	Strand	Location with Reference to Other Genes
misc_1	14,254–14,383	−	antisense to 3′ end of g048
misc_2	17,805–18,199	+	antisense to 5′ end of g055 and 3′ end of g056
misc_3	32,500–32,820	−	antisense to 5′ end of g077
misc_4	61,640–61,936	+	intragenic region between g099 and g100
misc_5	91,090–91,192	−	antisense to middle part of g144
misc_6	103,380–103,720	+	antisense to g157 and 5′ end of g156
misc_7	148,610–148,960	+	antisense to 5′ end of g207
misc_8	160,970–161,280	+	antisense to middle part of g230
misc_9	217,379–217,553	+	antisense to middle part of g295
misc_10	242,633–242,957	−	antisense to middle part of g326

**Table 2:** Selected bacterial genes differentially expressed between the negative control and the late time points of phiR1-37 infection.

Gene	Protein Names	Log2FC
**Membrane Proteins**		
***Y11_28711***	P pilus assembly/Cpx signaling pathway, periplasmic inhibitor	5.18
***Y11_27571***	Putative inner membrane protein	3.71
***Y11_38011***	UPF0391 membrane protein Y11_38011	2.56
***Y11_12421***	Putative inner membrane protein	2.34
***Y11_36571***	Membrane protein	2.30
***Y11_25791***	Integral membrane protein TerC	2.07
***Y11_00031***	Putative permease PerM (=YfgO)	1.98
***Y11_17401***	Outer membrane protein X	1.70
***Y11_23901***	Conserved putative membrane protein	−2.12
***Y11_12601***	Putative membrane protein YPO2012	−2.27
**Transporters**		
***Y11_29791***	Phosphate ABC transporter, periplasmic phosphate-binding protein PstS	5.33
***Y11_38431***	Na(+)/H(+) antiporter NhaA (Sodium/proton antiporter NhaA)	4.72
***Y11_14731***	Mg(2+) transport ATPase protein C	3.63
***Y11_29801***	Phosphate transport system permease protein PstC	3.59
***Y11_07561***	Putative ABC sugar transporter	2.70
***Y11_26421***	Uncharacterized ABC transporter, permease component YrbE	1.99
***Y11_43381***	Phosphate transport system permease protein PstC	1.70
***Y11_29811***	Phosphate transport system permease protein PstA	1.68
***Y11_00591***	Sialic acid transporter (Permease) NanT	−2.18
**Stress response Proteins**		
***Y11_08711***	Phage shock protein A	3.51
***Y11_08721***	Phage shock protein B	2.55
***Y11_08731***	Phage shock protein C	2.41
***Y11_08741***	Phage shock protein D	2.09
***Y11_04271***	Cold shock protein	3.10
***Y11_04291***	Cold shock protein CspB	2.96
***Y11_27291***	Cold shock protein CspG	1.97
***Y11_27301***	Cold shock protein CspG	1.87
***Y11_10461***	Osmotically inducible lipoprotein B	4.24
***Y11_38001***	Osmotically inducible protein OsmY	3.06
**Transcriptional Regulators**		
***Y11_38441***	Transcriptional activator NhaR	3.90
***Y11_21031***	Phosphate regulon transcriptional regulatory protein PhoB (SphR)	3.73
***Y11_22431***	Transcriptional regulator, ArsR family	2.67
***Y11_10431***	Transcriptional regulatory protein YciT	−2.44
***Y11_21251***	Transcriptional regulator, GntR family	−3.66
**Other**		
***Y11_34281***	RNA polymerase sigma factor	2.30
***Y11_28241***	Regulator of ribonuclease activity A	2.26
***Y11_21021***	Phosphate regulon sensor protein PhoR (SphS) (EC 2.7.13.3)	1.69

## Supplementary Materials

The following are available online at http://www.mdpi.com/1999-4915/8/4/111/s1. **Figure S1**: The Bioanalyzer electropherograms of total RNA isolated from the bacteria at different time points post-infection, **Figure S2**: The time-wise degradation of the host RNA during phage φR1-37 infection, **Table S1**: The number of reads that mapped to YeO3-R1 and phiR1-37 genomes, **Figure S3**: Principal component analysis (PCA) graph presenting the correlation between all the samples used in this study, **Table S2**: Expression pattern of phiR1-37 genes and their promoters, **Figure S4**. High resolution image of Figure 3A. **Figure S5**: The conservation of phage phiR1-37 promoters generated using the Meme-Suite, **Table S3**: Bacterial genes differentially expressed between the early time points of phiR1-37 infection (2–5 min) and the negative control, **Table S4**: Bacterial genes differentially expressed between the late time points of phiR1-37 infection (28–49 min) and the negative control, **Table S5**: Bacterial genes differentially expressed between the late and the early time points of phiR1-37 infection, **Figure S6**: The nucleotide metabolism.

## Abbreviations

The following abbreviations are used in this manuscript:

asRNA	antisense RNA
LB	lysogeny broth
log2FC	log2 value of the fold change
LPS	lipopolysaccharide
MOI	multiplicity of infection
ncRNA	non-coding RNA
OC	outer core
PFU	plaque forming units
RNAP	RNA polymerase
sRNA	small RNA
TC	Total Count
TGR	Total Gene Reads
