# Supplementary material for: RNA-Sequencing Reveals the Progression of Phage-Host Interactions between φR1-37 and Yersinia enterocolitica
Source: Viruses. 2016 Apr 22;8(4):111. doi: 10.3390/v8040111 (PMC4848604; doi:10.3390/v8040111)
Supplement: Supplementary file 1 [file viruses-08-00111-s001.docx]

Supplementary material

RNA-sequencing reveals the progression of phage-host interactions between φR1-37 and *Yersinia enterocolitica*

**Katarzyna Leskinen^1^, Bob G. Blasdel^2^, Rob Lavigne^2^ and Mikael Skurnik^1,3*^**

**^1^Department of Bacteriology and Immunology, Medicum, and Research Programs Unit, Immunobiology, University of Helsinki, Finland,**

**^2^Division of Gene Technology, Katholieke Universiteit Leuven, Leuven, Belgium,**

**^3^Division of Clinical Microbiology, Helsinki University Hospital, HUSLAB, Helsinki, Finland.**

***Address for correspondence:**

Mikael Skurnik

Department of Bacteriology and immunology

P.O.Box 21 (Haartmaninkatu 3)

FIN-00014 UNIVERSITY OF HELSINKI

FINLAND

tel: +358-2491 26464

fax: +358-2941 26382

[mikael.skurnik@helsinki.fi](mailto:mikael.skurnik@helsinki.fi)

**
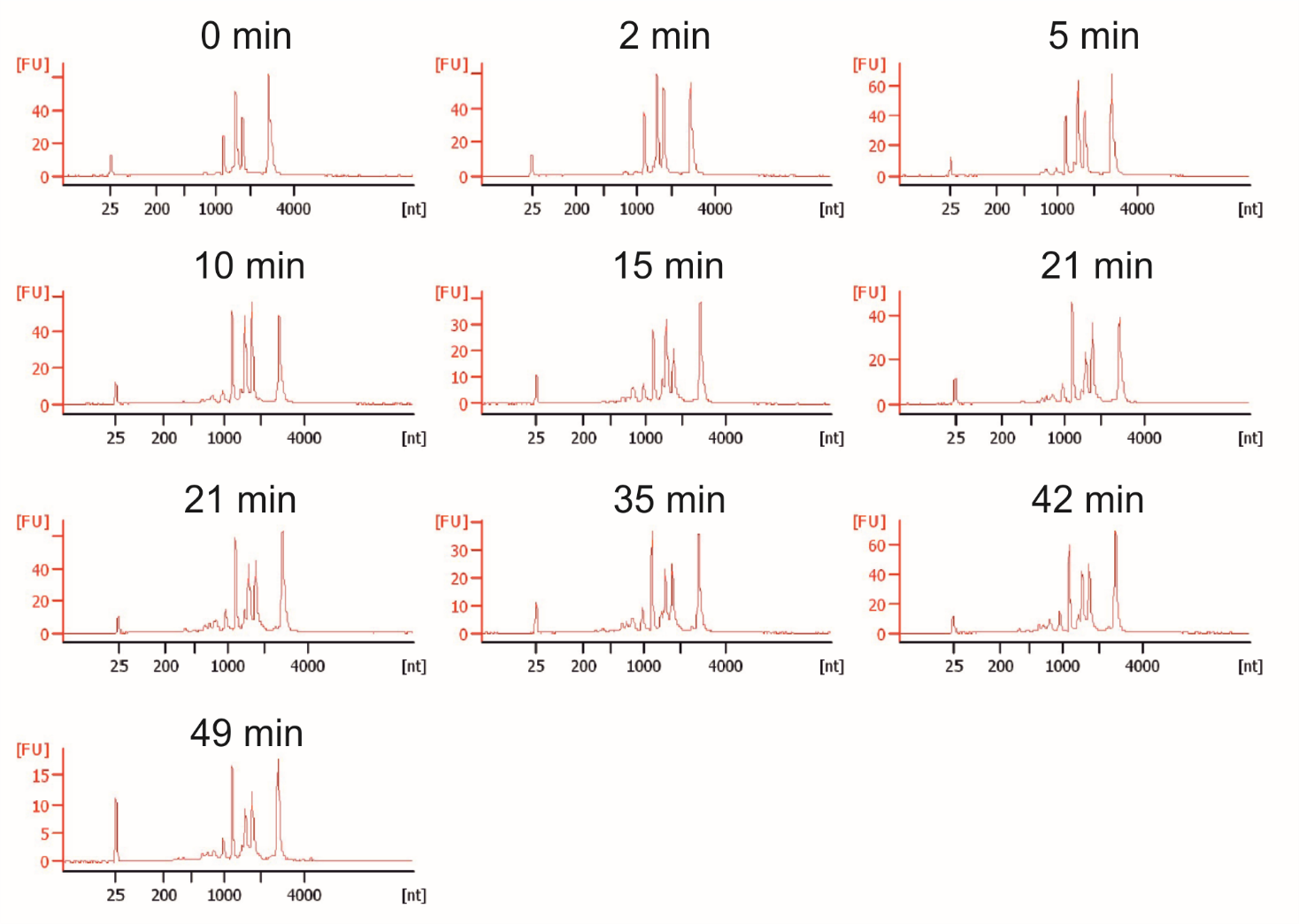
**

**Figure S1**. The Bioanalyzer electropherograms of total RNA isolated from the bacteria at different time points post-infection. The fluorescence peaks correspond to the following rRNA species: 5S rRNA (~120 nt), the 5’-end of 23S rRNA (~1170 nt), 16S rRNA (~1550 nt), 3’-end of 23S rRNA (~1750 nt) and the unprocessed 23’ rRNA (~3000 nt).


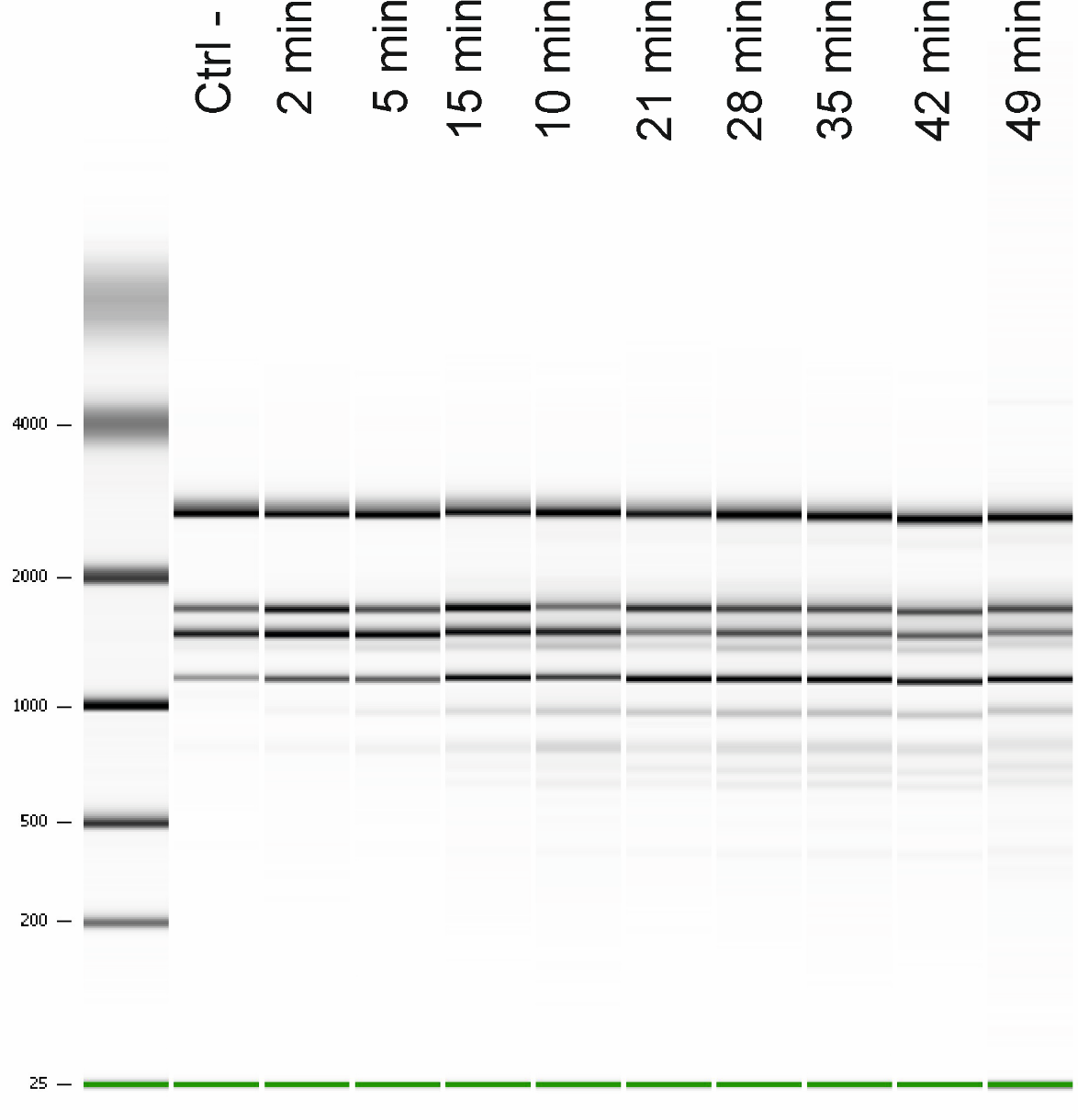


**Figure S2**. The time-wise degradation of the host RNA during phage φR1-37 infection. Bioanalyzer electrophoresis gel of total RNA isolated at different time points post-infection from the *Y. enterocolitica* YeO3-R1 bacteria infected with φR1-37. The main bands correspond to the following rRNA species: the processed 5’-end of 23S rRNA (~1170 nt), 16S rRNA (~1550 nt), the processed 3’-end of 23S rRNA (~1750 nt) and the unprocessed 23’ rRNA (~3000 nt).

**Table S1.** The number of reads that mapped to YeO3-R1 and phiR1-37 genomes.

|  |  | 0 | 2 | 5 | 10 | 15 | 21 | 28 | 35 | 42 | 49 |
| --- | --- | --- | --- | --- | --- | --- | --- | --- | --- | --- | --- |
| YeO3-R1 | For | 26934351 | 37439308 | 42238612 | 50805406 | 55501414 | 42091290 | 36977128 | 40415200 | 31389718 | 28508234 |
|  | Rev | 1239013 | 14269304 | 17463946 | 21232882 | 22406104 | 16370366 | 15359196 | 16836186 | 13675780 | 11710184 |
| phiR1-37 | For | 0 | 1433960 | 2991642 | 5257096 | 9785532 | 6142394 | 6285770 | 6513766 | 5549724 | 5839018 |
|  | Rev | 0 | 313132 | 609006 | 1355684 | 1417798 | 1356498 | 1493474 | 1604622 | 1319276 | 1658156 |

**
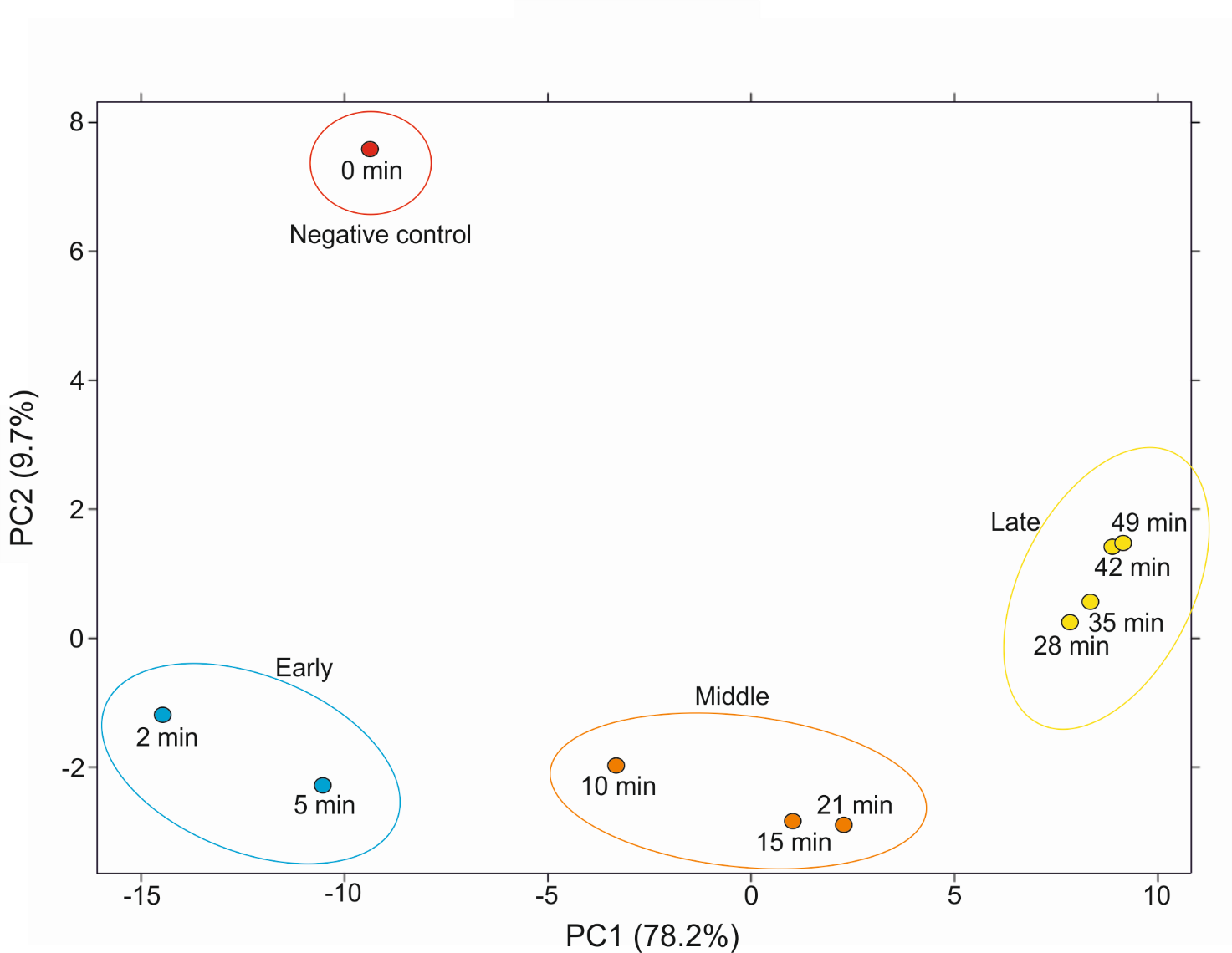
**

**Figure S3.** Principal component analysis (PCA) graph presenting the correlation between all the samples used in this study. Note, that the negative control was taken from the same flask immediately prior to infection.

**Figure S4**. Click [here](Leskinen_Viruses_Fig%203A_SI.tif) for High resolution image of **Figure 3A**. The progression of φR1-37 genome transcription during the infection cycle. The number of reads aligning to every 250 bp fragment of both strands of the phage genome was plotted for each time point. The intensity of the color of the curves from grey to black indicate the consecutive time points.

**Table S2.** Expression pattern of phiR1-37 genes and their promoters.

| Gene name | Strand | Functional Group | Ortholog, function, protein family, comments | Expression pattern | Mean TGR | | |
| --- | --- | --- | --- | --- | --- | --- | --- |
|  |  |  |  |  | **early** | **middle** | **late** |
| *g001* | **+** |  |  | EARLY | 129,91 | 70,22 | 50,32 |
| *g002* | **+** |  |  | NO | 240,38 | 190,95 | 195,17 |
| *g003* | **+** |  |  | NO | 303,01 | 206,31 | 229,72 |
| *g004* | **+** |  |  | EARLY | 13,08 | 6,78 | 1,13 |
| *g005* | **+** |  |  | EARLY | 12,28 | 3,79 | 2,27 |
| *g006* | **+** |  |  | NO | 250,50 | 192,07 | 265,74 |
| *g007* | **+** |  |  | EARLY | 17,86 | 10,00 | 6,52 |
| *g008* | **+** |  |  | EARLY | 20,60 | 8,22 | 5,86 |
| *g009* | **+** |  |  | EARLY | 74,57 | 50,91 | 33,16 |
| *g010* | **+** |  |  | EARLY | 11,01 | 4,21 | 1,49 |
| → | **PHIRE promoter (verified by RNA-seq)** | | | | | | |
| *g011* | **+** |  |  | EARLY | 78,72 | 36,01 | 11,38 |
| *g012* | **+** |  |  | EARLY | 90,07 | 37,17 | 23,19 |
| *g013* | **+** |  |  | EARLY | 569,89 | 254,15 | 193,52 |
| *g014* | **+** |  |  | EARLY | 476,22 | 241,64 | 179,20 |
| *g015* | **+** |  |  | EARLY | 248,56 | 115,57 | 98,83 |
| *g016* | **+** |  |  | EARLY | 95,26 | 54,63 | 22,54 |
| *g017* | **+** |  |  | EARLY | 3595,67 | 2156,26 | 1792,51 |
| *g018* | **+** |  |  | EARLY | 497,64 | 267,15 | 258,26 |
| *g019* | **+** |  |  | EARLY | 203,59 | 127,97 | 63,63 |
| *g020* | **+** |  |  | EARLY | 502,27 | 325,61 | 162,48 |
| *g021* | **+** |  |  | EARLY | 44,76 | 28,56 | 9,97 |
| *g022* | **+** |  |  | EARLY | 323,70 | 246,65 | 111,11 |
| *g023* | **+** |  |  | EARLY | 64,58 | 36,74 | 23,04 |
| *g024* | **+** |  |  | EARLY | 51,23 | 40,44 | 9,77 |
| *g025* | **+** |  |  | EARLY | 51,76 | 39,15 | 14,72 |
| *g026* | **+** |  |  | NO | 1130,04 | 1002,33 | 1076,14 |
| *g027* | **+** |  |  | EARLY | 220,81 | 209,79 | 47,43 |
| *g028* | **+** |  |  | EARLY | 57,28 | 47,81 | 18,27 |
| → | **PHIRE promoter** (verified by RNA-seq) | | | | | | |
| *g029* | **+** |  |  | EARLY | 210,51 | 119,25 | 50,63 |
| *g030* | **+** |  |  | EARLY | 1112,53 | 589,17 | 455,07 |
| *g031* | **+** |  |  | EARLY | 2121,51 | 1471,29 | 1077,92 |
| *g032* | **+** |  |  | EARLY | 972,44 | 524,43 | 365,19 |
| *g033* | **+** |  |  | NO | 1714,12 | 1134,27 | 1035,78 |
| *g034* | **+** |  |  | NO | 4635,52 | 3677,49 | 3709,61 |
| *g035* | **+** |  |  | EARLY | 163,44 | 98,12 | 65,83 |
| *g036* | **+** |  |  | NO | 1695,98 | 1314,73 | 1183,92 |
| *g037* | **+** |  |  | EARLY | 291,07 | 178,63 | 46,14 |
| *g038* | **+** |  |  | NO | 1214,71 | 914,80 | 788,13 |
| *g039* | **+** |  |  | EARLY | 82,47 | 62,87 | 23,83 |
| *g040* | **+** |  |  | EARLY | 529,67 | 336,12 | 124,27 |
| *g041* | **+** |  |  | EARLY | 307,33 | 257,81 | 115,69 |
| *g042* | **+** |  |  | EARLY | 840,32 | 501,03 | 354,05 |
| *g043* | **+** |  |  | EARLY | 114,70 | 55,15 | 17,33 |
| *g044* | **+** |  |  | MIDDLE | 22,96 | 34,91 | 9,65 |
| *g045* | **+** | virion structural protein | | NO | 1300,72 | 1325,65 | 1571,42 |
| *g046* | **+** | virion structural protein | Peptidoglycan-recognition protein-LC isoform LCA | NO | 568,20 | 577,77 | 892,29 |
| *g047* | **+** | virion structural protein | | NO | 281,96 | 242,37 | 423,27 |
| *g048* | **+** | virion structural protein | | NO | 950,17 | 793,56 | 1248,41 |
| *g049* | **-** | ATPase | ATP-ase. Pfam: PhoH-like protein (PF02562) | NO | 1146,93 | 1068,10 | 1676,76 |
| *g050* | **-** |  |  | NO | 27,37 | 32,40 | 23,84 |
| *g051* | **-** |  |  | NO | 132,30 | 108,54 | 137,05 |
| *g052* | **-** |  |  | NO | 1132,49 | 1203,65 | 1495,49 |
| *g053* | **-** |  |  | NO | 128,61 | 131,72 | 181,81 |
| *g054* | **-** |  |  | EARLY | 38,40 | 29,28 | 9,24 |
| *g055* | **-** |  |  | NO | 23798,42 | 28073,45 | 28325,80 |
| *g056* | **-** | nucleotide metabolism | Guanylate kinase, Pfam: guanylate kinase (PF00625) | NO | 16375,77 | 18331,02 | 17452,30 |
| *g057* | **-** |  |  | NO | 12254,39 | 11182,20 | 9126,95 |
| *g058* | **-** |  |  | NO | 14562,33 | 15634,97 | 15675,23 |
| *g059* | **-** |  |  | NO | 41627,79 | 34718,79 | 27781,23 |
| ← | **PHIRE promoter (verified by RNA-seq)** | | | | | | |
| → | **PHIRE promoter (verified by RNA-seq)** | | | | | | |
| *g060* | **+** | nucleotide metabolism | Phosphohydrolase, Pfam: NUDIX (PF00293) | NO | 856,58 | 754,41 | 1143,46 |
| *g061* | **+** | ATPase | ATPase, Pfam: AAA_23 (PF13476) | NO | 5738,63 | 6378,63 | 9495,95 |
| *g062* | **+** |  |  |  | 1,97 | 9,77 | 3,11 |
| → | **PHIRE promoter (verified by RNA-seq)** | | | | | | |
| *g063* | **+** |  |  | NO | 32863,39 | 34890,22 | 27613,36 |
| → | **PHIRE promoter (verified by RNA-seq)** | | | | | | |
| *g064* | **+** |  |  | LATE | 425,49 | 635,58 | 1118,46 |
| *g065* | **+** |  |  | MIDDLE | 11,73 | 20,18 | 7,57 |
| *g066* | **+** |  |  | LATE | 461,21 | 951,93 | 1781,27 |
| *g067* | **+** |  |  |  | 0,00 | 0,40 | 0,00 |
| *g068* | **+** | nucleotide metabolism | Phosphoribosyltransferase, Pfam: Pribosyltran_N (PF13793) + Pribosyltran (PF00156) | LATE | 2078,02 | 5273,56 | 11746,22 |
| *g069* | **+** | DNA replication and repair | HNH homing endonuclease | LATE | 760,14 | 2001,01 | 4087,57 |
| *g070* | **+** | nucleotide metabolism | Nicotinate phosphoribosyltransferase, Pfam: NAPRTase (PF04095) | LATE | 3355,81 | 7315,84 | 16767,97 |
| *g071* | **+** |  |  |  | 4,25 | 6,68 | 9,81 |
| *g072* | **+** |  |  | LATE | 334,21 | 472,77 | 963,42 |
| → | **PHIRE promoter (verified by RNA-seq)** | | | | | | |
| *g073* | **+** |  |  | LATE | 300,19 | 542,41 | 705,08 |
| *g074* | **+** | virion structural protein | | LATE | 546,32 | 1095,81 | 2312,42 |
| *g075* | **+** |  |  | LATE | 81,65 | 138,76 | 210,39 |
| *g076* | **+** |  |  | LATE | 158,71 | 242,78 | 498,34 |
| → | **PHIRE promoter (verified by RNA-seq)** | | | | | | |
| *g077* | **+** | nucleotide metabolism | Lincosamide antibiotic adenylyltransferase LinA | NO | 2553,68 | 2471,27 | 2489,83 |
| → | **PHIRE promoter (verified by RNA-seq)** | | | | | | |
| *g078* | **+** |  |  | EARLY | 19789,55 | 10245,80 | 6406,71 |
| *g079* | **-** | virion structural protein | Prohead core protein protease Pfam: Peptidase_U9 (PF03420) | NO | 715,63 | 491,47 | 776,26 |
| *g080* | **-** |  |  | NO | 613,16 | 440,44 | 451,73 |
| *g081* | **-** | virion structural protein | | NO | 1165,10 | 840,71 | 1219,00 |
| ← | **Sigma promoter** | | |  |  |  |  |
| → | **Sigma promoter** | | |  |  |  |  |
| *g082* | **+** | virion structural protein | | NO | 531,84 | 403,24 | 528,28 |
| *g083* | **+** | virion structural protein | Tail tape measure protein, transglycolase, Pfam: SLT (PF01464) | NO | 9312,35 | 6651,92 | 10661,88 |
| *g084* | **+** | lysis protein | Muramoyl peptidase, Pfam: Peptidase_M15_3 (PF05291) | NO | 325,76 | 264,64 | 329,68 |
| *g085* | **+** | virion structural protein | | NO | 1532,26 | 1177,29 | 1536,94 |
| *g086* | **+** | DNA replication and repair | Holliday junction resolvase, endonuclease, Pfam: RusA (PF05866) | NO | 650,16 | 539,54 | 844,16 |
| *g087* | **+** |  |  | NO | 1373,36 | 1095,54 | 1449,50 |
| *g088* | **+** | virion structural protein | | EARLY | 13,93 | 6,86 | 7,67 |
| *g089* | **+** | virion structural protein | Peptidase | NO | 934,21 | 825,17 | 1006,38 |
| *g090* | **+** |  |  | NO | 743,23 | 595,25 | 1129,98 |
| *g091* | **+** |  |  | NO | 377,84 | 424,85 | 581,86 |
| *g092* | **+** |  |  | LATE | 494,47 | 749,40 | 1299,51 |
| → | **PHIRE promoter (verified by RNA-seq)** | | | | | | |
| *g093* | **+** |  |  | LATE | 884,70 | 1918,58 | 2861,45 |
| *g094* | **-** | virion structural protein | | NO | 32,82 | 20,50 | 35,10 |
| ← | **Sigma promoter**  (verified by RNA-seq) | | | | | | |
| → | **Sigma promoter (verified by RNA-seq)** | | |  |  |  |  |
| *g095* | **+** | uncharacterized | Uncharacterized protein | NO | 229,67 | 190,99 | 199,79 |
| → | **PHIRE promoter (verified by RNA-seq)** | | |  |  |  |  |
| *g096* | **+** | DNA replication and repair | Reverse transcriptase, Pfam: RVT_1 (PF00078) | LATE | 3558,73 | 8146,01 | 16614,50 |
| *g097* | **+** | ATPase | ATP-dependent Clp protease, Pfam: CLP_protease (PF00574) | NO | 721,98 | 719,87 | 1069,33 |
| *g098* | **+** |  |  | LATE | 505,04 | 542,81 | 1015,86 |
| *g099* | **-** | virion structural protein | Structural protein. Putative RNA-polymerase b-subunit | NO | 5501,10 | 4306,92 | 6771,73 |
| ← | **Sigma promoter** | | |  |  |  |  |
| → | **Sigma promoter** | | |  |  |  |  |
| *g100* | **+** | nucleotide metabolism | Non-canonical purine NTP pyrophosphatase, Pfam: rdgB/Ham1p_like (PF01725) | LATE | 1208,49 | 3568,27 | 7037,89 |
| *g101* | **+** | virion structural protein | Polyadenylate-binding protein 1; RNA-binding | LATE | 2929,52 | 21421,23 | 62946,05 |
| *g102* | **+** | RNAP and RNA interaction | Putative DNA-directed RNA polymerase, b'-subunit | LATE | 3203,88 | 4195,50 | 7628,39 |
| *g103* | **+** | nucleotide metabolism | CMP/dCMP deaminase, zinc-binding, Pfam: dCMP_cyt_deam_1 (PF00383) | LATE | 338,83 | 829,35 | 1953,37 |
| *g104* | **+** |  |  |  | 8,20 | 5,19 | 7,09 |
| *g105* | **+** |  |  | LATE | 26,15 | 28,55 | 50,11 |
| → | **PHIRE promoter (verified by RNA-seq)** | | | |  |  |  |
| *g106* | **+** | diverse | BH1478 protein; unknown function | | 0,00 | 0,20 | 0,00 |
| → | **PHIRE promoter (verified by RNA-seq)** | | |  |  |  |  |
| *g107* | **+** | DNA replication and repair | HNH endonuclease, Pfam: HNH-3 (PF13392) | NO | 870,84 | 925,31 | 1021,01 |
| *g108* | **+** |  |  | NO | 52,00 | 42,53 | 37,36 |
| *g109* | **+** |  |  |  | 0,00 | 1,11 | 0,00 |
| *g110* | **+** |  |  | LATE | 8,52 | 16,91 | 23,34 |
| *g111* | **+** |  |  | LATE | 393,88 | 493,86 | 814,39 |
| → | **PHIRE promoter (verified by RNA-seq)** | | |  |  |  |  |
| *g112* | **+** |  |  | NO | 2243,34 | 2421,94 | 1996,51 |
| *g113* | **+** |  |  | EARLY | 288,24 | 288,46 | 127,41 |
| *g114* | **+** |  |  | EARLY | 1382,26 | 1079,71 | 759,18 |
| *g115* | **+** |  |  | EARLY | 110,44 | 100,85 | 35,75 |
| *g116* | **-** |  |  |  | 0,00 | 0,81 | 0,83 |
| ← | **Sigma promoter** | | |  |  |  |  |
| → | **Sigma promoter** | | |  |  |  |  |
| *g117* | **+** |  |  |  | 3,91 | 1,51 | 1,16 |
| → | **PHIRE promoter (verified by RNA-seq)** | | |  |  |  |  |
| *g118* | **+** |  |  | NO | 34486,25 | 27326,99 | 22549,57 |
| *g119* | **+** |  |  | EARLY | 4788,73 | 3509,39 | 2286,42 |
| *g120* | **+** |  |  | NO | 2692,23 | 2541,90 | 2115,33 |
| *g121* | **+** | DNA replication and repair | Type III restriction enzyme, Pfam: ResIII (PF04851) | NO | 2411,83 | 1918,63 | 2942,95 |
| → | **PHIRE promoter (verified by RNA-seq)** | | |  |  |  |  |
| *g122* | **+** |  |  | NO | 14830,82 | 12940,29 | 11776,06 |
| *g123* | **+** |  |  | NO | 13326,86 | 10833,55 | 8330,30 |
| *g124* | **+** |  |  | NO | 23025,53 | 19368,64 | 14975,86 |
| *g125* | **+** |  |  | NO | 20559,18 | 19330,18 | 17622,82 |
| *g126* | **+** |  |  | MIDDLE | 5367,29 | 5757,50 | 5038,87 |
| *g127* | **+** |  |  | EARLY | 48,82 | 41,36 | 5,18 |
| *g128* | **+** | virion structural protein | | NO | 3072,36 | 2670,96 | 3563,90 |
| *g129* | **+** | virion structural protein | RAN GTPase-activating protein 1 | LATE | 583,57 | 492,21 | 1499,45 |
| *g130* | **+** | virion structural protein | | LATE | 588,65 | 447,45 | 1488,15 |
| *g131* | **+** | virion structural protein | Bacterial DNA-binding protein, Pfam: Bac_DNA_binding (PF00216) | LATE | 118,28 | 72,41 | 659,61 |
| → | **PHIRE promoter (verified by RNA-seq)** | | |  |  |  |  |
| *g132* | **+** |  |  | EARLY | 390,84 | 221,49 | 146,84 |
| *g133* | **+** | virion structural protein | Gluconate kinase | NO | 220,03 | 160,04 | 291,76 |
| *g134* | **+** | virion structural protein | | LATE | 551,18 | 451,15 | 1078,56 |
| *g135* | **+** | virion structural protein | Major capsid protein sp46 precursor. Also detected from 28, 35, 43, 47, 94 and 102 kDa bands. | LATE | 943,17 | 674,77 | 2306,74 |
| → | **PHIRE promoter (verified by RNA-seq)** | | |  |  |  |  |
| *g136* | **+** |  |  | NO | 576,83 | 531,06 | 855,05 |
| → | **PHIRE promoter (unverified)** | | |  |  |  |  |
| *g137* | **+** |  |  | NO | 612,36 | 525,88 | 494,29 |
| *g138* | **+** |  |  | NO | 741,75 | 591,00 | 427,41 |
| *g139* | **+** |  |  | EARLY | 12027,21 | 7715,86 | 6263,36 |
| *g140* | **+** |  |  | EARLY | 3349,89 | 2279,58 | 1609,99 |
| *g141* | **+** |  |  | NO | 539,08 | 336,71 | 494,37 |
| *g142* | **+** | diverse | PAAR repeat containing protein, Pfam: PAAR_motif (PF05488) | NO | 47,71 | 26,56 | 42,82 |
| *g143* | **+** | DNA replication and repair | Terminase large subunit, helicases | NO | 767,61 | 701,69 | 727,15 |
| → | **PHIRE promoter (verified by RNA-seq)** | | |  |  |  |  |
| *g144* | **+** |  |  | NO | 196253,42 | 173593,56 | 171179,40 |
| *g145* | **+** | DNA replication and repair | DNA helicase, terminase large subunit, Pfam: Terminase_6 (PF03237) | NO | 2134,55 | 1578,56 | 2328,37 |
| *g146* | **+** | virion structural protein | | LATE | 574,17 | 451,56 | 768,68 |
| *g147* | **+** |  |  | LATE | 597,99 | 474,68 | 1489,50 |
| *g148* | **+** | virion structural protein | | NO | 355,50 | 255,10 | 474,97 |
| *g149* | **+** | virion structural protein | Tail sheath protein Pfam: Phage_Sheath_1 (PF04984) | LATE | 991,59 | 669,33 | 1626,41 |
| *g150* | **+** | virion structural protein | Structural protein sp31. N-terminal sequence. | NO | 430,34 | 308,00 | 588,39 |
| *g151* | **-** | DNA replication and repair | DNA mismatch endonuclease, Pfam: Vsr (PF03852) | LATE | 1336,23 | 2149,25 | 3947,14 |
| *g152* | **-** |  |  | LATE | 1840,31 | 6349,36 | 11413,21 |
| ← | **PHIRE promoter (verified by RNA-seq)** | | |  |  |  |  |
| *g153* | **-** |  |  | LATE | 26,51 | 31,85 | 51,67 |
| *g154* | **-** | uncharacterized | Uncharacterized protein | LATE | 1249,71 | 2178,34 | 4206,48 |
| *g155* | **-** |  |  | LATE | 38,47 | 35,55 | 73,08 |
| *g156* | **-** |  |  | LATE | 369,33 | 1066,36 | 2189,29 |
| *g157* | **-** |  |  | LATE | 30,91 | 96,46 | 185,20 |
| *g158* | **-** |  |  | LATE | 411,00 | 730,44 | 1260,76 |
| *g159* | **-** |  |  | LATE | 63,68 | 132,26 | 171,33 |
| ← | **PHIRE promoter (verified by RNA-seq)** | | |  |  |  |  |
| *g160* | **-** | virion structural protein | Head portal vertex protein | LATE | 2568,37 | 1977,49 | 3826,58 |
| *g161* | **-** | virion structural protein | | NO | 790,84 | 517,51 | 773,40 |
| *g162* | **-** | virion structural protein | | NO | 561,75 | 395,19 | 581,52 |
| *g163* | **-** | virion structural protein | | NO | 138,72 | 94,29 | 105,26 |
| *g164* | **-** | DNA replication and repair | DNA polymerase II | LATE | 644,13 | 866,29 | 1861,71 |
| *g165* | **-** |  |  |  | 0,34 | 0,40 | 0,00 |
| *g166* | **-** |  |  |  | 0,00 | 0,00 | 0,00 |
| *g167* | **-** | DNA replication and repair | DNA polymerase, Pfam: DNA_pol_B (PF00136) | LATE | 1850,70 | 2524,98 | 6130,29 |
| ← | **Sigma promoter** | | |  |  |  |  |
| → | **Sigma promoter** | | |  |  |  |  |
| *g168* | **+** | virion structural protein | | LATE | 376,52 | 272,52 | 678,83 |
| *g169* | **-** |  |  | NO | 1038,93 | 726,84 | 1176,22 |
| → | **PHIRE promoter (verified by RNA-seq)** | | |  |  |  |  |
| *g170* | **+** | DNA replication and repair | DNA polymerase, Pfam: DNA_pol_B (PF00136) | LATE | 2120,19 | 3392,48 | 7748,80 |
| *g171* | **+** | DNA replication and repair | Similarity to fR1-37 g230, a putative homing endonuclease | LATE | 1951,02 | 3115,61 | 6446,09 |
| *g172* | **+** |  |  |  | 2,32 | 1,70 | 0,33 |
| → | **PHIRE promoter (verified by RNA-seq)** | | |  |  |  |  |
| *g173* | **+** |  |  | NO | 23366,20 | 18146,61 | 15911,16 |
| *g174* | **+** | virion structural protein | | NO | 10010,66 | 9310,82 | 9825,08 |
| *g175* | **+** |  |  | LATE | 1071,09 | 1432,93 | 3512,15 |
| → | **PHIRE promoter (verified by RNA-seq)** | | | |  |  |  |
| *g176* | **+** | virion structural protein | | LATE | 2225,09 | 7276,97 | 17137,12 |
| *g177* | **+** |  |  | LATE | 811,39 | 1304,04 | 2679,88 |
| *g178* | **+** | virion structural protein | Structural protein, RNA polymerase b'-subunit | NO | 1624,31 | 1182,63 | 2175,25 |
| → | **PHIRE promoter (verified by RNA-seq)** | | |  |  |  |  |
| *g179* | **+** | uncharacterized | Uncharacterized protein | NO | 1355,31 | 1406,29 | 1857,61 |
| *g180* | **+** |  |  |  | 5,47 | 3,32 | 0,88 |
| *g181* | **+** |  |  |  | 0,00 | 5,24 | 0,00 |
| → | **PHIRE promoter (verified by RNA-seq)** | | |  |  |  |  |
| *g182* | **+** |  |  | NO | 1326,99 | 1093,68 | 845,75 |
| *g183* | **+** |  |  | NO | 1141,42 | 1097,72 | 1346,47 |
| *g184* | **+** |  |  | NO | 665,68 | 597,76 | 460,04 |
| *g185* | **+** |  |  | NO | 1207,46 | 1152,69 | 1127,23 |
| *g186* | **+** |  |  | NO | 537,53 | 739,84 | 667,30 |
| *g187* | **+** | DNA replication and repair | Phosphoesterase, Pfam: metallophos_2 (PF12850) | NO | 2876,55 | 3447,99 | 3923,77 |
| → | **PHIRE promoter (unverified)** | | |  |  |  |  |
| *g188* | **+** |  |  |  | 0,63 | 1,21 | 0,00 |
| *g189* | **+** |  |  | EARLY | 53,93 | 49,70 | 5,15 |
| *g190* | **+** | uncharacterized | Uncharacterized protein | NO | 32682,61 | 39000,08 | 41992,88 |
| *g191* | **+** | uncharacterized | Uncharacterized protein | NO | 13921,83 | 14581,91 | 16005,61 |
| *g192* | **+** |  |  | NO | 16260,95 | 20880,84 | 24415,82 |
| *g193* | **+** |  |  | NO | 5986,26 | 6365,53 | 6874,55 |
| → | **PHIRE promoter (unverified)** | | |  |  |  |  |
| *g194* | **+** |  |  | LATE | 3712,96 | 4747,68 | 7215,79 |
| *g195* | **+** |  |  | LATE | 969,80 | 1270,00 | 2162,24 |
| *g196* | **+** | virion structural protein | | NO | 2617,59 | 2183,09 | 3241,63 |
| *g197* | **-** | virion structural protein | | NO | 390,63 | 268,83 | 419,08 |
| *g198* | **-** | virion structural protein | | NO | 602,28 | 471,26 | 742,55 |
| *g199* | **-** | virion structural protein | Structural protein sp24 precursor. Also present in 22 and 24 kDa bands. | LATE | 320,74 | 227,93 | 1550,73 |
| *g200* | **-** |  |  |  | 3,53 | 2,32 | 0,33 |
| *g201* | **-** |  |  |  | 0,97 | 1,21 | 0,91 |
| *g202* | **-** |  |  | LATE | 318,52 | 240,28 | 635,02 |
| *g203* | **-** | virion structural protein | | NO | 320,34 | 254,26 | 495,48 |
| *g204* | **-** | virion structural protein | Probable protease HTPX homolog | NO | 930,06 | 587,58 | 1172,11 |
| *g205* | **-** | virion structural protein | | NO | 970,83 | 701,05 | 1110,06 |
| *g206* | **-** | virion structural protein | | NO | 986,67 | 662,97 | 1184,53 |
| *g207* | **-** | virion structural protein | | NO | 2119,91 | 1467,20 | 2555,75 |
| ← | **PHIRE promoter (verified by RNA-seq)** | | | |  |  |  |
| *g208* | **-** | virion structural protein | | NO | 1090,24 | 716,75 | 1550,96 |
| ← | **Sigma promoter** | | |  |  |  |  |
| → | **Sigma promoter** | | | |  |  |  |
| *g209* | **+** | virion structural protein | | NO | 1230,11 | 790,34 | 1600,20 |
| *g210* | **+** | virion structural protein | | LATE | 681,85 | 526,11 | 1173,35 |
| → | **PHIRE promoter (verified by RNA-seq)** | | |  |  |  |  |
| *g211* | **+** |  |  | EARLY | 32250,83 | 21799,50 | 16169,77 |
| *g212* | **+** | RNAP and RNA interaction | Ribonuclease H, Pfam RNase_H (PF00075) | NO | 916,68 | 698,41 | 624,96 |
| *g213* | **+** |  |  | NO | 20404,16 | 22223,63 | 21020,24 |
| *g214* | **+** |  |  | NO | 2239,96 | 3031,44 | 2840,74 |
| *g215* | **+** |  |  | NO | 2290,42 | 1964,41 | 1487,10 |
| *g216* | **+** |  |  | MIDDLE | 4076,72 | 4211,01 | 3650,49 |
| *g217* | **+** |  |  |  | 1,31 | 3,02 | 0,00 |
| *g218* | **+** |  |  | NO | 3548,01 | 2894,68 | 2614,72 |
| *g219* | **+** | diverse | Hypothetical protein rv5_Gp079 | EARLY | 3118,49 | 2390,45 | 1569,83 |
| *g220* | **+** |  |  | EARLY | 373,89 | 235,20 | 173,36 |
| *g221* | **+** |  |  | EARLY | 1045,51 | 715,13 | 378,84 |
| *g222* | **+** |  |  | MIDDLE | 164,95 | 171,44 | 28,38 |
| *g223* | **+** |  |  | EARLY | 270,09 | 165,17 | 36,34 |
| *g224* | **+** | uncharacterized | Uncharacterized protein | NO | 8328,23 | 7735,40 | 7306,74 |
| *g225* | **+** |  |  | NO | 6013,80 | 6454,21 | 7045,48 |
| *g226* | **+** |  |  | NO | 4835,15 | 4431,27 | 3936,01 |
| *g227* | **+** |  |  | EARLY | 488,01 | 434,40 | 216,71 |
| *g228* | **-** |  |  | LATE | 63,06 | 42,45 | 76,03 |
| *g229* | **-** | virion structural protein | PRE-mRNA processing protein PRP40 | NO | 112,10 | 84,38 | 165,52 |
| *g230* | **-** | DNA replication and repair | Putative homing endonuclease, similarity to fR1-37 Gp171 | NO | 1974,07 | 1750,75 | 3219,42 |
| *g231* | **-** | virion structural protein | Structural protein, DNA-directed RNA polymerase, b-subunit | NO | 1932,39 | 1472,78 | 2438,31 |
| *g232* | **-** |  |  |  | 0,34 | 0,60 | 0,00 |
| ← | **Sigma antisense promoter (unverified)** | | |  |  |  |  |
| → | **PHIRE promoter (unverified)** | | |  |  |  |  |
| *g233* | **-** | virion structural protein | | LATE | 381,12 | 294,34 | 965,96 |
| *g234* | **-** | virion structural protein | | NO | 2961,68 | 2443,19 | 4041,43 |
| *g235* | **-** |  |  | NO | 154,45 | 125,96 | 224,57 |
| *g236* | **-** | virion structural protein | NF-YC; histone-like PAIR | LATE | 147,33 | 112,58 | 210,63 |
| ← | **Sigma promoter** | | |  |  |  |  |
| → | **Sigma promoter** | | |  |  |  |  |
| *g237* | **+** | virion structural protein | Structural protein, putative Vsr/MutH/archaeal HJR family endonuclease | NO | 613,60 | 476,75 | 822,23 |
| *g238* | **+** |  |  | LATE | 339,05 | 279,89 | 521,04 |
| *g239* | **+** | virion structural protein | | NO | 88,65 | 74,45 | 101,28 |
| *g240* | **+** | virion structural protein | | LATE | 157,38 | 78,13 | 177,44 |
| *g241* | **+** |  |  | LATE | 1361,29 | 1013,44 | 4380,87 |
| *g242* | **+** |  |  |  | 8,60 | 1,01 | 9,19 |
| *g243* | **+** | diverse | CPN60(groel); chaperonin, chaperone, groel, HSP60 | LATE | 2356,38 | 1803,94 | 4771,68 |
| *g244* | **+** | virion structural protein | | LATE | 954,06 | 839,02 | 1926,94 |
| → | **PHIRE promoter (verified by RNA-seq)** | | |  |  |  |  |
| *g245* | **+** |  |  | LATE | 1854,04 | 3054,13 | 6148,45 |
| *g246* | **+** |  |  | LATE | 447,94 | 704,99 | 1498,18 |
| *g247* | **+** | DNA replication and repair | DNA primase | LATE | 543,66 | 1453,21 | 3480,19 |
| *g248* | **+** |  |  | LATE | 462,80 | 696,07 | 1122,12 |
| *g249* | **+** |  |  | LATE | 619,51 | 1504,74 | 3143,67 |
| *g250* | **+** | DNA replication and repair | Protein RECArecombinase A | LATE | 1579,55 | 3411,27 | 8043,42 |
| → | **PHIRE promoter (verified by RNA-seq)** | | |  |  |  |  |
| *g251* | **+** |  |  | EARLY | 10284,78 | 7002,97 | 6088,89 |
| → | **PHIRE promoter (verified by RNA-seq)** | | |  |  |  |  |
| *g252* | **+** |  |  | EARLY | 410,90 | 218,33 | 78,01 |
| *g253* | **+** |  |  | EARLY | 40520,17 | 29506,37 | 22668,63 |
| *g254* | **+** |  |  | EARLY | 3319,29 | 2368,12 | 1837,16 |
| *g255* | **+** |  |  | NO | 1806,98 | 1304,08 | 882,34 |
| *g256* | **+** |  |  | NO | 1534,18 | 1105,73 | 1020,43 |
| *g257* | **+** |  |  | EARLY | 2648,27 | 1899,26 | 1478,39 |
| *g258* | **+** |  |  | NO | 6087,20 | 4978,27 | 4085,81 |
| *g259* | **+** |  |  | NO | 3856,84 | 3498,03 | 3116,69 |
| *g260* | **+** |  |  | NO | 3073,45 | 2584,76 | 2374,47 |
| → | **PHIRE promoter (verified by RNA-seq)** | | |  |  |  |  |
| *g261* | **+** | RNAP and RNA interaction | DNA-directed RNA polymerase subunit beta | LATE | 3988,10 | 6704,78 | 13385,29 |
| → | **PHIRE promoter (verified by RNA-seq)** | | |  |  |  |  |
| *g262* | **+** |  |  | EARLY | 1126,75 | 641,35 | 275,51 |
| *g263* | **+** | uncharacterized | Uncharacterized protein | LATE | 697,93 | 485,24 | 949,77 |
| → | **PHIRE promoter (verified by RNA-seq)** | | |  |  |  |  |
| *g264* | **+** |  |  | LATE | 68,20 | 123,26 | 217,86 |
| *g265* | **+** | uncharacterized | Uncharacterized protein | LATE | 366,03 | 707,00 | 1196,81 |
| *g266* | **+** |  |  | LATE | 107,00 | 178,56 | 341,31 |
| *g267* | **+** | DNA replication and repair | DNA-ligase | LATE | 2474,68 | 4444,90 | 8336,36 |
| *g268* | **+** |  |  | LATE | 27,70 | 43,22 | 89,55 |
| *g269* | **+** | DNA replication and repair | Single-stranded DNA-binding protein | LATE | 97,23 | 201,96 | 402,53 |
| *g270* | **-** | virion structural protein | | NO | 1277,41 | 1166,67 | 1828,68 |
| *g271* | **-** | virion structural protein | | NO | 2040,13 | 1777,04 | 2733,21 |
| *g272* | **-** | virion structural protein | | NO | 1660,65 | 1294,80 | 2349,46 |
| *g273* | **-** |  |  | LATE | 111,26 | 435,56 | 950,46 |
| *g274* | **-** | RNAP and RNA interaction | DNA-directed RNA polymerase subunit beta' | LATE | 1581,75 | 2554,00 | 4593,13 |
| ← | **PHIRE promoter (verified by RNA-seq)** | | | |  |  |  |
| → | **PHIRE promoter (verified by RNA-seq)** | | | |  |  |  |
| *g275* | **+** | virion structural protein | | NO | 453,76 | 412,41 | 643,33 |
| → | **PHIRE promoter (verified by RNA-seq)** | | |  |  |  |  |
| *g276* | **+** | uncharacterized | Uncharacterized protein | NO | 1419,63 | 1223,85 | 1346,68 |
| *g277* | **-** | uncharacterized | Uncharacterized protein. Similarity to Gp282 of fR1-37. | LATE | 83,96 | 69,53 | 146,82 |
| *g278* | **-** |  |  | NO | 1192,34 | 987,78 | 1369,76 |
| *g279* | **-** |  |  | NO | 542,29 | 423,92 | 491,01 |
| *g280* | **-** | virion structural protein | oxidative stress DPS DNA binding | NO | 208,00 | 145,04 | 251,92 |
| *g281* | **-** | virion structural protein | | NO | 255,90 | 178,80 | 276,35 |
| *g282* | **-** | uncharacterized | Uncharacterized protein. Similarity to Gp278 of fR1-37. | LATE | 217,38 | 182,68 | 315,65 |
| → | **PHIRE promoter (unverified)** | | |  |  |  |  |
| → | **PHIRE promoter (verified by RNA-seq)** | | |  |  |  |  |
| *g283* | **+** |  |  | NO | 117448,15 | 106103,93 | 81909,60 |
| *g284* | **+** | RNAP and RNA interaction | Putative RNA-binding protein | EARLY | 1670,69 | 1421,65 | 902,50 |
| *g285* | **-** | virion structural protein | Type VI secretion system component | NO | 1092,03 | 662,08 | 1099,73 |
| ← | **Sigma promoter** | | |  |  |  |  |
| → | **PHIRE promoter (verified by RNA-seq)** | | |  |  |  |  |
| *g286* | **+** | DNA replication and repair | Exonuclease V alpha chain, Pfam: AAA_30 (PF13604), UvrD_C_2 (PF13538) | LATE | 1993,87 | 3029,59 | 5659,62 |
| *g287* | **+** | DNA replication and repair | Homing endonuclease, Pfam: HNH_3 (PF13392) | LATE | 734,79 | 1272,36 | 2659,05 |
| *g288* | **+** |  |  |  | 0,00 | 0,00 | 0,00 |
| *g289* | **-** | lysis protein | Endo-type membrane-bound lytic murein transglycosylase | NO | 366,37 | 260,44 | 417,31 |
| *g290* | **-** |  |  | NO | 25,08 | 13,14 | 26,76 |
| *g291* | **-** |  |  | NO | 88,15 | 67,63 | 99,66 |
| *g292* | **-** |  |  | LATE | 547,99 | 428,40 | 987,78 |
| *g293* | **-** | uncharacterized | Uncharacterized protein | NO | 504,14 | 379,28 | 720,66 |
| *g294* | **-** | virion structural protein | Kelch-like protein 12 | NO | 4402,31 | 3080,02 | 5603,21 |
| *g295* | **-** | virion structural protein | Kelch-like ECH-associated protein 1 | NO | 4488,10 | 3147,76 | 5140,65 |
| *g296* | **-** | diverse | Kelch-like protein 12 | NO | 4280,12 | 3051,10 | 5886,15 |
| *g297* | **-** | virion structural protein | Caudovirales tail fibre assembly protein, Pfam: Caudo_TAP (PF02413) | NO | 802,46 | 552,48 | 1205,71 |
| *g298* | **-** | virion structural protein | Phage tail collar protein, Pfam: Collar (PF07484) | NO | 1953,78 | 1350,22 | 2741,92 |
| *g299* | **-** | virion structural protein | Conserved hypothetical phage tail fiber protein | NO | 113,39 | 81,28 | 116,91 |
| *g300* | **-** | virion structural protein | Phage tail collar domain protein | NO | 1791,87 | 1144,38 | 2182,14 |
| *g301* | **-** |  |  | NO | 613,64 | 378,35 | 607,21 |
| *g302* | **-** |  |  | NO | 51,48 | 39,40 | 46,16 |
| ← | **Sigma promoter** | | |  |  |  |  |
| → | **PHIRE promoter (verified by RNA-seq)** | | |  |  |  |  |
| *g303* | **+** |  |  | NO | 5849,41 | 5045,08 | 4328,09 |
| → | **PHIRE promoter (verified by RNA-seq)** | | |  |  |  |  |
| *g304* | **+** | ATPase | ATPase, Pfam: AAA_5 (PF07728) | NO | 301555,11 | 296648,96 | 276993,76 |
| *g305* | **+** | diverse | Uncharacterized protein with a domain of unknown function, Pfam: DUF2201 (PF09967) | NO | 132619,79 | 129167,24 | 111641,18 |
| → | **PHIRE promoter (verified by RNA-seq)** | | |  |  |  |  |
| *g306* | **+** |  |  | MIDDLE | 59,69 | 111,45 | 95,95 |
| *g307* | **+** |  |  | LATE | 598,82 | 1816,81 | 3280,48 |
| *g308* | **+** | Toxin-antidote | Antidote protein, Pfam: HTH_3 (PF01381) | LATE | 189,45 | 453,09 | 935,21 |
| *g309* | **+** | Toxin-antidote | Plasmid maintenance system antidote protein, Pfam: HTH_3 (PF01381) | LATE | 77,61 | 203,86 | 468,00 |
| → | **PHIRE promoter (verified by RNA-seq)** | | |  |  |  |  |
| *g310* | **+** |  |  | LATE | 4,25 | 13,36 | 14,43 |
| *g311* | **+** | DNA replication and repair | DNA gyrase B, Pfam: HATpase_c (PF02518), DNA_gyrase_B (PF00204) | LATE | 1086,54 | 2288,17 | 5081,93 |
| *g312* | **+** |  |  |  | 0,00 | 1,21 | 0,00 |
| *g313* | **+** | DNA replication and repair | VSR endonuclease | LATE | 363,80 | 519,51 | 872,58 |
| *g314* | **+** | DNA replication and repair | DNA gyrase subunit B | LATE | 1705,29 | 4822,80 | 12054,24 |
| *g315* | **+** |  |  |  | 0,00 | 0,00 | 0,00 |
| → | **PHIRE promoter (verified by RNA-seq)** | | |  |  |  |  |
| *g316* | **+** |  |  |  | 3,91 | 4,33 | 0,61 |
| *g317* | **+** |  |  | EARLY | 45,97 | 25,82 | 7,34 |
| *g318* | **+** |  |  | EARLY | 242,49 | 152,66 | 107,47 |
| *g319* | **+** |  |  | EARLY | 301,31 | 183,85 | 164,44 |
| *g320* | **+** |  |  | EARLY | 626,01 | 576,16 | 370,17 |
| *g321* | **+** |  |  | EARLY | 455,22 | 293,32 | 219,66 |
| *g322* | **+** |  |  | EARLY | 579,01 | 418,84 | 299,14 |
| *g323* | **+** |  |  | NO | 1299,68 | 1267,50 | 1106,50 |
| *g324* | **+** |  |  | MIDDLE | 94,00 | 97,90 | 49,86 |
| *g325* | **+** |  |  | NO | 9378,64 | 11656,89 | 15259,52 |
| → | **PHIRE promoter (verified by RNA-seq)** | | | |  |  |  |
| *g326* | **+** | virion structural protein | | NO | 182601,18 | 187011,15 | 187747,53 |
| → | **PHIRE promoter (verified by RNA-seq)** | | | |  |  |  |
| *g327* | **+** | DNA replication and repair | DNA topoisomerase IV subunit A, Pfam: DNA_topoisoIV (PF00521) | | 0,34 | 3,19 | 3,81 |
| *g328* | **+** | DNA replication and repair | homing endonuclease I-DMOI | LATE | 711,73 | 1137,73 | 2630,14 |
| *g329* | **+** | DNA replication and repair | DNA gyrase A, Pfam: DNA_topoisoIV (PF00521), four repeats of DNA_gyrase_A_c (PF03989) | LATE | 2547,79 | 6157,62 | 15607,30 |
| → | **PHIRE promoter (verified by RNA-seq)** | | |  |  |  |  |
| *g330* | **+** |  |  | NO | 9704,51 | 9220,16 | 8398,94 |
| *g331* | **+** | lysis protein | Endo-type membrane-bound lytic murein transglycosylase | EARLY | 115600,21 | 79794,25 | 59040,64 |
| *g332* | **+** |  |  | NO | 121169,10 | 116455,58 | 100919,01 |
| *g333* | **-** | virion structural protein | | NO | 369,26 | 262,56 | 429,36 |
| ← | **PHIRE promoter (verified by RNA-seq)** | | |  |  |  |  |
| → | **PHIRE promoter (verified by RNA-seq)** | | |  |  |  |  |
| *g334* | **+** | DNA replication and repair | Exonuclease | LATE | 892,72 | 1261,18 | 2469,00 |
| *g335* | **+** | DNA replication and repair | DNAB replication FORK helicase | LATE | 933,35 | 1296,44 | 2566,24 |
| *g336* | **+** | DNA replication and repair | VSR endonuclease | LATE | 455,01 | 668,09 | 1352,71 |
| *g337* | **+** | DNA replication and repair | DNAB-like replicative helicase | LATE | 1277,74 | 2617,23 | 5096,84 |
| *g338* | **+** |  |  |  | 8,41 | 5,39 | 9,31 |
| *g339* | **+** |  |  | EARLY | 278,54 | 119,42 | 67,15 |
| *g340* | **+** |  |  | EARLY | 64,61 | 44,06 | 22,31 |
| *g341* | **+** |  |  | EARLY | 43,17 | 36,16 | 13,72 |
| *g342* | **+** |  |  | EARLY | 323,85 | 184,93 | 150,21 |
| *g343* | **+** |  |  | NO | 399,15 | 321,50 | 240,57 |
| *g344* | **+** |  |  | EARLY | 1077,62 | 704,37 | 402,57 |
| *g345* | **+** |  |  | EARLY | 523,41 | 374,29 | 262,21 |
| *g346* | **+** |  |  | EARLY | 289,96 | 193,30 | 142,99 |
| *g347* | **+** |  |  | EARLY | 159,08 | 129,05 | 82,45 |
| *g348* | **+** |  |  | EARLY | 143,49 | 106,74 | 48,05 |
| *g349* | **+** |  |  | EARLY | 233,64 | 164,69 | 99,20 |
| *g350* | **+** |  |  | EARLY | 1116,81 | 779,91 | 615,64 |
| *g351* | **+** |  |  | NO | 1801,68 | 1287,33 | 1163,40 |
| *g352* | **+** |  |  | EARLY | 253,41 | 157,22 | 105,25 |
| *g353* | **+** |  |  | EARLY | 141,40 | 71,46 | 54,10 |
| *g354* | **+** |  |  | EARLY | 85,60 | 52,56 | 42,39 |
| *g355* | **+** |  |  | EARLY | 367,77 | 211,36 | 206,48 |
| *g356* | **+** |  |  | MIDDLE | 31,45 | 37,39 | 3,91 |
| *g357* | **+** |  |  | EARLY | 85,69 | 36,22 | 10,68 |
| *g358* | **+** |  |  | EARLY | 74,16 | 35,82 | 13,80 |
| *g359* | **+** |  |  | EARLY | 28,74 | 15,24 | 3,66 |
| *g360* | **+** |  |  | EARLY | 260,85 | 141,17 | 74,92 |
| *g361* | **+** |  |  | EARLY | 117,96 | 67,17 | 27,19 |
| *g362* | **+** |  |  | EARLY | 55,89 | 26,67 | 17,79 |
| *g363* | **+** |  |  | EARLY | 61,44 | 45,85 | 23,32 |
| *g364* | **+** |  |  | EARLY | 114,72 | 50,21 | 39,58 |
| *g365* | **+** |  |  | EARLY | 77,25 | 36,57 | 14,64 |
| *g366* | **+** |  |  | EARLY | 164,23 | 85,90 | 82,65 |
| *g367* | **+** |  |  |  | 5,07 | 3,22 | 0,28 |

**
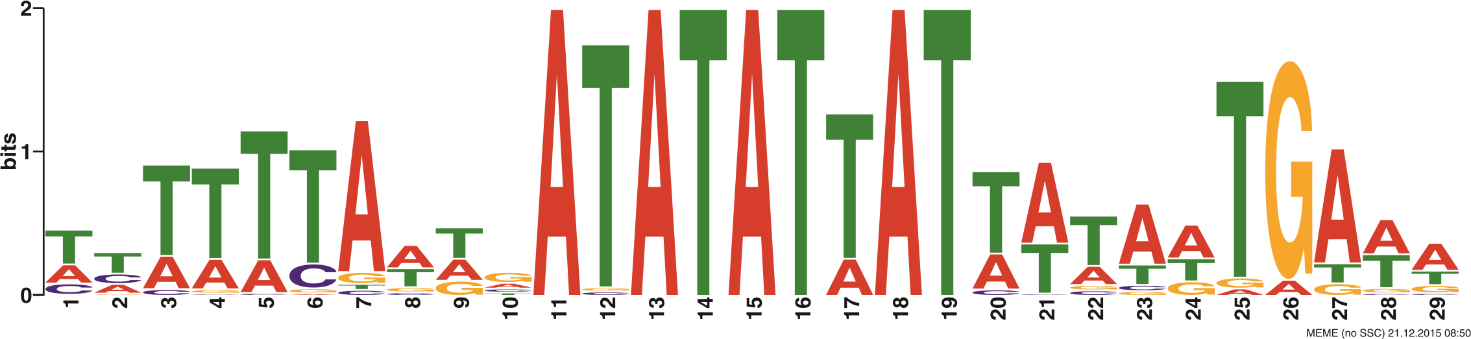
**

**Figure S5.** The conservation of phage phiR1-37 promoters generated using the Meme-Suite (http://meme-suite.org).

**Table S3.** Bacterial genes differentially expressed between the early time points of phiR1-37 infection (2 - 5 min) and the negative control. Log2FC value was calculated as the log_2_ of the ratio between the mean read counts of the negative control and phage infected samples.

| Name | Protein names | | Log2FC | |
| --- | --- | --- | --- | --- |
| *Y11_38431* | Na(+)/H(+) antiporter NhaA (Sodium/proton antiporter NhaA) | | 3.14 | |
| *Y11_37711* | Glycerol dehydrogenase (EC 1.1.1.6) | | 3.14 | |
| *Y11_36571* | Membrane protein | | 1.86 | |
| *Y11_28961* | 50S ribosomal protein L33 | | 1.76 | |
| *Y11_29681* | ATP synthase protein I | | 1.68 | |
| *Y11_04281* | Uncharacterized protein | | 1.61 | |
| *Y11_14731* | Mg(2+) transport ATPase protein C | | 1.57 | |
| *Y11_06561* | Gene D protein | | -1.50 | |
| *Y11_19521* | Uncharacterized protein | | -1.51 | |
| *Y11_06231* | Uncharacterized protein | | -1.51 | |
| *Y11_00541* | Putative N-acetylmannosamine-6-phosphate 2-epimerase (EC 5.1.3.9) (ManNAc-6-P epimerase) | | -1.52 | |
| *Y11_26621* | Protein containing transglutaminase-like domain, putative cysteine protease | | -1.52 | |
| *Y11_32291* | Type IV pilus biogenesis protein PilN | | -1.52 | |
| *Y11_12831* | Phage tail protein | | -1.52 | |
| *Y11_03821* | O-methyltransferase involved in polyketide biosynthesis | | -1.52 | |
| *Y11_02771* | Putative transport protein | | -1.52 | |
| *Y11_11071* | Hnr protein | | -1.53 | |
| *Y11_26831* | Soluble cytochrome b562 | | -1.53 | |
| *Y11_05991* | Uncharacterized protein | | -1.54 | |
| *Y11_21891* | Putative lipoprotein | | -1.55 | |
| *Y11_43241* | Probable lipid kinase YegS-like (EC 2.7.-.-) (EC 2.7.1.-) | | -1.55 | |
| *Y11_22011* | Response regulator of the LytR/AlgR family | | -1.55 | |
| *Y11_13711* | Putative aminoacrylate hydrolase RutD (EC 3.5.1.-) (Aminohydrolase) | | -1.56 | |
| *Y11_11441* | Spovr-like protein | | -1.56 | |
| *Y11_30211* | Putative transport protein Y11_30211 | | -1.57 | |
| *Y11_38121* | Colicin type 7 (Cja) | | -1.57 | |
| *Y11_05701* | Transposase for IS1668 | | -1.58 | |
| *Y11_00661* | Macrolide export ATP-binding/permease protein MacB | | -1.58 | |
| *Y11_07251* | Uncharacterized protein | | -1.59 | |
| *Y11_32301* | Type IV pilus biogenesis protein PilO | | -1.59 | |
| *Y11_00441* | Transcriptional regulator, LysR family | | -1.59 | |
| *Y11_01181* | Putative exported protein | | -1.59 | |
| *Y11_07681* | Methylmalonyl-CoA mutase (EC 5.4.99.2) | | -1.59 | |
| *Y11_37621* | Autoinducer 2 (AI-2) ABC transport system, fused AI2 transporter subunits and ATP-binding component | | -1.60 | |
| *Y11_22471* | Cytochrome c551 peroxidase (EC 1.11.1.5) | | -1.60 | |
| *Y11_00671* | Macrolide export ATP-binding/permease protein MacB | | -1.61 | |
| *Y11_07321* | Sigma-fimbriae uncharacterized paralogous subunit | | -1.61 | |
| *Y11_19021* | Uncharacterized protein | | -1.62 | |
| *Y11_17041* | Hydrogenase-4 component D | | -1.62 | |
| *Y11_05711* | Transposase for IS1668 | | -1.62 | |
| *Y11_15091* | Exonuclease SbcC | | -1.63 | |
| *Y11_17181* | Mgl repressor and galactose ultrainduction factor GalS, HTH-type transcriptional regulator | | -1.64 | |
| *Y11_24581* | Type IV prepilin peptidase TadV/CpaA | | -1.64 | |
| *Y11_01291* | Putative transport | | -1.64 | |
| *Y11_17851* | Phosphonate ABC transporter permease protein phnE1 | | -1.64 | |
| *Y11_30641* | Xylose activator XylR (AraC family) | | -1.65 | |
| *Y11_38811* | Tryptophanase (EC 4.1.99.1) | | -1.65 | |
| *Y11_00301* | Uncharacterized protein | | -1.66 | |
| *Y11_30421* | Virulence associated protein C | | -1.67 | |
| *Y11_28651* | Uncharacterized protein | | -1.67 | |
| *Y11_36871* | UPF0213 protein Y11_36871 | | -1.68 | |
| *Y11_20201* | Protein containing PTS-regulatory domain | | -1.68 | |
| *Y11_10511* | Putative exported protein YPO2521 | | -1.68 | |
| *Y11_09621* | Uncharacterized protein | | -1.68 | |
| *Y11_36261* | Upf0379 protein yjfY | | -1.69 | |
| *Y11_02861* | Putative ATP-binding protein | | -1.70 | |
| *Y11_16161* | Propanediol utilization polyhedral body protein PduB | | -1.70 | |
| *Y11_30411* | Virulence-associated protein vagC | | -1.70 | |
| *Y11_40701* | Prepilin peptidase dependent protein A | | -1.71 | |
| *Y11_13451* | Major tail tube protein | | -1.71 | |
| *Y11_34391* | Alpha-related fimbriae chaperone 2 | | -1.72 | |
| *Y11_06921* | Putative cytochrome | | -1.72 | |
| *Y11_37061* | Phosphonates transport ATP-binding protein PhnL | | -1.72 | |
| *Y11_41811* | Uncharacterized protein | | -1.72 | |
| *Y11_21081* | UPF0345 protein Y11_21081 | | -1.73 | |
| *Y11_21901* | Ribosome hibernation protein YfiA | | -1.75 | |
| *Y11_41791* | Nickel transport ATP-binding protein nikE2 | | -1.75 | |
| *Y11_37111* | Phnh protein | | -1.75 | |
| *Y11_19461* | Transcriptional regulator, LysR family | | -1.76 | |
| *Y11_22371* | Putative transcriptional regulator of sorbose uptake and utilization genes | | -1.77 | |
| *Y11_43231* | PTS system, glucitol/sorbitol-specific IIC component (EC 2.7.1.69) | | -1.78 | |
| *Y11_30681* | Phage capsid and scaffold protein | | -1.78 | |
| *Y11_39221* | Type IV pilin PilA | | -1.78 | |
| *Y11_08531* | Uncharacterized protein | | -1.80 | |
| *Y11_39341* | Transcriptional regulator of AraC family,enterobactin-dependent, predicted | | -1.81 | |
| *Y11_12931* | Phage tail fiber protein | | -1.82 | |
| *Y11_01561* | Uncharacterized protein | | -1.82 | |
| *Y11_37441* | Transposase | | -1.82 | |
| *Y11_35751* | Radical SAM family protein HutW, similar to coproporphyrinogen III oxidase, oxygen-independent, associated with heme uptake | | -1.84 | |
| *Y11_24041* | Hydrogenase-2 operon protein hybA | | -1.84 | |
| *Y11_37081* | Phnj protein | | -1.88 | |
| *Y11_15371* | [citrate [pro-3S]-lyase] ligase (EC 6.2.1.22) | | -1.88 | |
| *Y11_06121* | Uncharacterized protein | | -1.89 | |
| *Y11_10901* | Uncharacterized protein | | -1.90 | |
| *Y11_12181* | ISPsy11, transposase OrfA | | -1.92 | |
| *Y11_22711* | Uncharacterized protein | | -1.93 | |
| *Y11_35761* | Threonine dehydratase, catabolic (EC 4.3.1.19) | | -1.93 | |
| *Y11_10361* | Putative formate dehydrogenase oxidoreductase protein | | -1.94 | |
| *Y11_15001* | Protein MtfA (Mlc titration factor A) | | -1.95 | |
| *Y11_10441* | Uncharacterized protein | | -1.96 | |
| *Y11_34461* | sn-glycerol-3-phosphate transport system permease protein UgpA | | -1.97 | |
| *Y11_16131* | Propanediol utilization transcriptional activator | | -1.98 | |
| *Y11_39741* | Deoxyguanosinetriphosphate triphosphohydrolase (dGTP triphosphohydrolase) (dGTPase) (EC 3.1.5.1) | | -1.99 | |
| *Y11_19871* | Uncharacterized protein | | -2.02 | |
| *Y11_41701* | Transcriptional regulator, AraC family | | -2.05 | |
| *Y11_04321* | Uncharacterized protein | | -2.05 | |
| *Y11_26141* | Uncharacterized protein | | -2.19 | |
| *Y11_06541* | Integrase |  | | -2.19 |
| *Y11_05031* | Putative insecticidal toxin complex | | -2.22 | |
| *Y11_05781* | Uncharacterized protein | | -2.28 | |
| *Y11_05901* | Uncharacterized protein | | -2.29 | |
| *Y11_10521* | Benzoate transport protein | | -2.31 | |
| *Y11_09201* | Ribokinase (EC 2.7.1.15) | | -2.33 | |
| *Y11_30941* | Ypjf toxin protein | | -2.37 | |
| *Y11_14701* | Universal stress protein C | | -2.39 | |
| *Y11_14901* | Glucose-1-phosphate adenylyltransferase (EC 2.7.7.27) (ADP-glucose pyrophosphorylase) (ADP-glucose synthase) | | -2.58 | |
| *Y11_24051* | Uptake hydrogenase small subunit (EC 1.12.99.6) | | -2.60 | |
| *Y11_00591* | Sialic acid transporter (Permease) NanT | | -2.85 | |
| *Y11_32431* | Nitrite reductase [NAD(P)H] large subunit (EC 1.7.1.4) | | -2.96 | |
| *Y11_37931* | Uncharacterized protein | | -3.06 | |
| *Y11_12601* | Putative membrane protein YPO2012 | | -3.36 | |
| *Y11_21251* | Transcriptional regulator, GntR family | | -3.38 | |

**Table S4.** Bacterial genes differentially expressed between the late time points of phiR1-37 infection (28 - 49 min) and the negative control. Log2FC value was calculated as the log_2_ of the ratio between the mean read counts of the negative control and phage infected samples.

| Name | Protein names | | Log2FC |
| --- | --- | --- | --- |
| *Y11_33811* | Transposase | | 5.91 |
| *Y11_29791* | Phosphate ABC transporter, periplasmic phosphate-binding protein PstS | | 5.33 |
| *Y11_28711* | P pilus assembly/Cpx signaling pathway, periplasmic inhibitor/zinc-resistance associated protein | | 5.18 |
| *Y11_36501* | Putative exported protein YPO3518 | | 4.81 |
| *Y11_38431* | Na(+)/H(+) antiporter NhaA (Sodium/proton antiporter NhaA) | | 4.72 |
| *Y11_37711* | Glycerol dehydrogenase (EC 1.1.1.6) | | 4.40 |
| *Y11_10461* | Osmotically inducible lipoprotein B | | 4.24 |
| *Y11_00861* | Putative exported protein | | 4.16 |
| *Y11_04281* | Uncharacterized protein | | 4.04 |
| *Y11_38441* | Transcriptional activator NhaR | | 3.90 |
| *Y11_21031* | Phosphate regulon transcriptional regulatory protein PhoB (SphR) | | 3.73 |
| *Y11_27571* | Putative inner membrane protein | | 3.71 |
| *Y11_33481* | Rhs family protein | | 3.66 |
| *Y11_14731* | Mg(2+) transport ATPase protein C | | 3.63 |
| *Y11_29801* | Phosphate transport system permease protein PstC | | 3.59 |
| *Y11_08711* | Phage shock protein A | | 3.51 |
| *Y11_01281* | Uncharacterized protein | | 3.48 |
| *Y11_15891* | Uncharacterized protein | | 3.48 |
| *Y11_42831* | Uncharacterized protein | | 3.27 |
| *Y11_05511* | Putative exported protein | | 3.26 |
| *Y11_20341* | Uncharacterized protein | | 3.20 |
| *Y11_32171* | Uncharacterized protein | | 3.18 |
| *Y11_04271* | Cold shock proteins | | 3.10 |
| *Y11_18901* | Uncharacterized protein ybfE | | 3.10 |
| *Y11_38001* | Osmotically inducible protein OsmY | | 3.06 |
| *Y11_04291* | Cold shock protein CspB | | 2.96 |
| *Y11_24541* | Cobalt-zinc-cadmium resistance protein CzcA Cation efflux system protein CusA | | 2.81 |
| *Y11_00241* | Uncharacterized protein | | 2.78 |
| *Y11_09891* | Uncharacterized protein | | 2.77 |
| *Y11_07931* | Uncharacterized protein | | 2.74 |
| *Y11_20021* | Uncharacterized protein | | 2.70 |
| *Y11_07561* | Putative ABC sugar transporter | | 2.70 |
| *Y11_22431* | Transcriptional regulator, ArsR family | | 2.67 |
| *Y11_37721* | Phosphoenolpyruvate-dihydroxyacetone phosphotransferase, dihydroxyacetone binding subunit DhaK (EC 2.7.1.121) | | 2.63 |
| *Y11_41931* | Uncharacterized protein | | 2.60 |
| *Y11_38011* | UPF0391 membrane protein Y11_38011 | | 2.56 |
| *Y11_32131* | Uncharacterized protein | | 2.55 |
| *Y11_08721* | Phage shock protein B | | 2.55 |
| *Y11_06011* | Cytolethal distending toxin subunit B | | 2.53 |
| *Y11_05751* | Isocitrate dehydrogenase [NADP] (EC 1.1.1.42) | | 2.48 |
| *Y11_10241* | Uncharacterized protein | | 2.47 |
| *Y11_08731* | Phage shock protein C | | 2.41 |
| *Y11_28961* | 50S ribosomal protein L33 | | 2.38 |
| *Y11_23181* | 3-phenylpropionate dioxygenase ferredoxin subunit | | 2.36 |
| *Y11_07811* | Uncharacterized protein | | 2.36 |
| *Y11_21161* | Putative exported protein YPO3518 | | 2.36 |
| *Y11_12421* | Putative inner membrane protein | | 2.34 |
| *Y11_36571* | Membrane protein | | 2.30 |
| *Y11_34281* | RNA polymerase sigma factor | | 2.30 |
| *Y11_33181* | Uncharacterized protein | | 2.26 |
| *Y11_28241* | Regulator of ribonuclease activity A | | 2.26 |
| *Y11_20361* | Glycoprotein-polysaccharide metabolism | | 2.22 |
| *Y11_02941* | Uncharacterized protein | | 2.22 |
| *Y11_28331* | Ferredoxin--NADP(+) reductase (EC 1.18.1.2) | | 2.21 |
| *Y11_37731* | Phosphoenolpyruvate-dihydroxyacetone phosphotransferase, ADP-binding subunit DhaL (EC 2.7.1.121) | | 2.17 |
| *Y11_01391* | Cytochrome c heme lyase subunit CcmH | | 2.16 |
| *Y11_19641* | Integrase |  | 2.14 |
| *Y11_20321* | Putative cytoplasmic protein | | 2.13 |
| *Y11_08741* | Phage shock protein D | | 2.09 |
| *Y11_25791* | Integral membrane protein TerC | | 2.07 |
| *Y11_25041* | Dihydroneopterin aldolase (EC 4.1.2.25) | | 2.07 |
| *Y11_28551* | Tail fiber assembly protein | | 2.04 |
| *Y11_36681* | Preprotein translocase subunit SecG | | 2.00 |
| *Y11_26421* | Uncharacterized ABC transporter, permease component YrbE | | 1.99 |
| *Y11_00031* | Putative permease PerM (=YfgO) | | 1.98 |
| *Y11_00231* | Uncharacterized protein | | 1.98 |
| *Y11_27291* | Cold shock protein CspG | | 1.97 |
| *Y11_39401* | Uncharacterized protein | | 1.94 |
| *Y11_41911* | Putative exported protein | | 1.94 |
| *Y11_10211* | Electron transport complex protein RnfA (Nitrogen fixation protein RnfA) | | 1.93 |
| *Y11_42101* | SsrA-binding protein | | 1.93 |
| *Y11_10871* | Acyl-CoA thioesterase YciA, involved in membrane biogenesis | | 1.92 |
| *Y11_33591* | Fimbrial protein | | 1.89 |
| *Y11_27301* | Cold shock protein CspG | | 1.87 |
| *Y11_27871* | Transposase | | 1.86 |
| *Y11_08371* | Transcriptional regulator, TetR family | | 1.84 |
| *Y11_01271* | Alkylphosphonate utilization operon protein PhnA | | 1.84 |
| *Y11_17221* | Putative MFS Superfamily transporter | | 1.82 |
| *Y11_35621* | Yoeb toxin protein | | 1.82 |
| *Y11_42161* | Uncharacterized protein | | 1.82 |
| *Y11_26921* | Protein AaeX | | 1.81 |
| *Y11_01401* | Lipoprotein | | 1.80 |
| *Y11_35201* | Secretion system apparatus SsaC | | 1.80 |
| *Y11_24451* | Transposase | | 1.79 |
| *Y11_00801* | Putative exported protein | | 1.78 |
| *Y11_28301* | Multidrug resistance protein D | | 1.77 |
| *Y11_36311* | Putative exported protein | | 1.77 |
| *Y11_20501* | Uncharacterized protein | | 1.74 |
| *Y11_26911* | p-hydroxybenzoic acid efflux pump subunit AaeA (pHBA efflux pump protein A) | | 1.73 |
| *Y11_28161* | Met repressor (Met regulon regulatory protein MetJ) | | 1.73 |
| *Y11_35871* | Fxsa protein | | 1.73 |
| *Y11_18471* | Protein TolB | | 1.72 |
| *Y11_32411* | Nitrite transporter NirC | | 1.72 |
| *Y11_18141* | Putative amino acid transporter | | 1.72 |
| *Y11_13031* | ISPsy4, transposition helper protein | | 1.72 |
| *Y11_31611* | Cob(I)alamin adenosyltransferase (EC 2.5.1.17) | | 1.71 |
| *Y11_21561* | Methylthioribose kinase (MTR kinase) (EC 2.7.1.100) | | 1.70 |
| *Y11_17391* | Putative signal peptide protein | | 1.70 |
| *Y11_12671* | Uncharacterized protein | | 1.70 |
| *Y11_17401* | Outer membrane protein X | | 1.70 |
| *Y11_43381* | Phosphate transport system permease protein PstC | | 1.70 |
| *Y11_21021* | Phosphate regulon sensor protein PhoR (SphS) (EC 2.7.13.3) | | 1.69 |
| *Y11_21221* | Flagellar brake protein YcgR (Cyclic di-GMP binding protein YcgR) | | 1.68 |
| *Y11_29811* | Phosphate transport system permease protein PstA | | 1.68 |
| *Y11_20331* | Haemolysin expression modulating protein | | 1.67 |
| *Y11_22241* | Anaerobic dimethyl sulfoxide reductase chain C | | 1.66 |
| *Y11_10861* | Probable intracellular septation protein A | | 1.65 |
| *Y11_19381* | Serine/threonine protein phosphatase (EC 3.1.3.16) | | 1.64 |
| *Y11_03681* | Putrescine transport system permease protein PotI | | 1.64 |
| *Y11_33471* | Uncharacterized protein | | 1.64 |
| *Y11_26431* | Uncharacterized ABC transporter, ATP-binding protein YrbF | | 1.64 |
| *Y11_25931* | Inner membrane protein YqjE | | 1.63 |
| *Y11_33161* | Uncharacterized protein | | 1.62 |
| *Y11_22231* | Putative oxidoreductase component of anaerobic dehydrogenases Functional role page for Chaperone protein TorD | | 1.61 |
| *Y11_35941* | Quaternary ammonium compound-resistance protein sugE | | 1.60 |
| *Y11_19631* | Inorganic pyrophosphatase/exopolyphosphatase | | 1.60 |
| *Y11_40331* | General secretion pathway protein D | | 1.59 |
| *Y11_30381* | Transporter, LysE family | | 1.59 |
| *Y11_40181* | Phage DNA binding protein | | 1.58 |
| *Y11_24341* | Flagellar basal body rod protein FlgB | | 1.58 |
| *Y11_29681* | ATP synthase protein I | | 1.57 |
| *Y11_06261* | Putative manganese efflux pump MntP | | 1.57 |
| *Y11_10451* | L-ribulose-5-phosphate 4-epimerase (EC 5.1.3.4) | | 1.54 |
| *Y11_00711* | Putative two-component system sensor kinase | | 1.53 |
| *Y11_25091* | DNA primase | | 1.53 |
| *Y11_09701* | Uncharacterized protein | | 1.52 |
| *Y11_09811* | UPF0482 protein Y11_09811 | | 1.51 |
| *Y11_07021* | Biofilm PGA synthesis auxiliary protein PgaD | | 1.50 |
| *Y11_23171* | Putative metalloprotease yggG | | 1.50 |
| *Y11_41811* | Uncharacterized protein | | -1.50 |
| *Y11_13821* | Ferrous iron transport peroxidase EfeB | | -1.50 |
| *Y11_10061* | Arabinose operon regulatory protein | | -1.51 |
| *Y11_00791* | Putative cytoplasmic protein | | -1.51 |
| *Y11_41571* | Glutaredoxin-like protein NrdH, required for reduction of Ribonucleotide reductase class Ib | | -1.51 |
| *Y11_13261* | Phage major capsid protein | | -1.52 |
| *Y11_42151* | Probable inorganic polyphosphate/ATP-NAD kinase (Poly(P)/ATP NAD kinase) (EC 2.7.1.23) | | -1.52 |
| *Y11_25771* | Putative metal-dependent hydrolase | | -1.52 |
| *Y11_00561* | N-acetylmannosamine kinase (EC 2.7.1.60) (ManNAc kinase) (N-acetyl-D-mannosamine kinase) | | -1.53 |
| *Y11_17311* | Putative transmembrane protein | | -1.54 |
| *Y11_23301* | UPF0301 protein Y11_23301 | | -1.54 |
| *Y11_27721* | Maltose/maltodextrin ABC transporter, substrate binding periplasmic protein MalE | | -1.54 |
| *Y11_08001* | Bifunctional polymyxin resistance protein ArnA | | -1.54 |
| *Y11_01571* | Aspartate racemase (EC 5.1.1.13) | | -1.54 |
| *Y11_35671* | ABC-type hemin transport system, ATPase component | | -1.56 |
| *Y11_10471* | Transposase | | -1.56 |
| *Y11_27111* | Maf-like protein Y11_27111 | | -1.56 |
| *Y11_01561* | Uncharacterized protein | | -1.57 |
| *Y11_11421* | D-amino acid dehydrogenase small subunit (EC 1.4.99.1) | | -1.57 |
| *Y11_00601* | Putative sialic acid transporter (Sialic acid permease) | | -1.58 |
| *Y11_38601* | Ribosomal RNA small subunit methyltransferase A (EC 2.1.1.182) (16S rRNA (adenine(1518)-N(6)/adenine(1519)-N(6))-dimethyltransferase) (16S rRNA dimethyladenosine transferase) (16S rRNA dimethylase) (S-adenosylmethionine-6-N', N'-adenosyl(rRNA) dimethyltransferase) | | -1.58 |
| *Y11_11171* | 5-methyl-dCTP pyrophosphohydrolase | | -1.59 |
| *Y11_01051* | Ferric iron ABC transporter, ATP-binding protein | | -1.59 |
| *Y11_00541* | Putative N-acetylmannosamine-6-phosphate 2-epimerase (EC 5.1.3.9) (ManNAc-6-P epimerase) | | -1.59 |
| *Y11_14001* | RNA polymerase sigma factor | | -1.60 |
| *Y11_29321* | Putative haloacid dehalogenase-like hydrolase STY3852 | | -1.61 |
| *Y11_13451* | Major tail tube protein | | -1.62 |
| *Y11_26831* | Soluble cytochrome b562 | | -1.63 |
| *Y11_32851* | Bacterioferritin-associated ferredoxin | | -1.64 |
| *Y11_11071* | Hnr protein | | -1.64 |
| *Y11_16491* | Ribose ABC transport system, ATP-binding protein RbsA | | -1.65 |
| *Y11_21871* | Protein acetyltransferase | | -1.66 |
| *Y11_06701* | Putative ROK-family transcriptional regulator | | -1.66 |
| *Y11_24381* | Transposase | | -1.67 |
| *Y11_03881* | Virulence factor VirK | | -1.68 |
| *Y11_33961* | Transcriptional regulator, TetR family | | -1.68 |
| *Y11_07731* | Manganese ABC transporter, periplasmic-binding protein SitA | | -1.69 |
| *Y11_28261* | Glycerol uptake facilitator protein | | -1.70 |
| *Y11_07991* | Undecaprenyl-phosphate 4-deoxy-4-formamido-L-arabinose transferase (EC 2.7.8.30) (Undecaprenyl-phosphate Ara4FN transferase) | | -1.70 |
| *Y11_33901* | Putative lipoprotein | | -1.70 |
| *Y11_11021* | Alcohol dehydrogenase Acetaldehyde dehydrogenase (EC 1.1.1.1) (EC 1.2.1.10) | | -1.70 |
| *Y11_17171* | Galactose/methyl galactoside ABC transport system, D-galactose-binding periplasmic protein MglB | | -1.71 |
| *Y11_40571* | Galactose operon repressor, GalR-LacI family of transcriptional regulators | | -1.71 |
| *Y11_09151* | Uncharacterized protein | | -1.72 |
| *Y11_32191* | Phosphoenolpyruvate carboxykinase [ATP] (PEP carboxykinase) (PEPCK) (EC 4.1.1.49) (Phosphoenolpyruvate carboxylase) | | -1.72 |
| *Y11_39741* | Deoxyguanosinetriphosphate triphosphohydrolase (dGTP triphosphohydrolase) (dGTPase) (EC 3.1.5.1) | | -1.73 |
| *Y11_12361* | Iron-chelator utilization protein | | -1.73 |
| *Y11_06781* | Ribulose-5-phosphate 4-epimerase and related epimerases and aldolases | | -1.74 |
| *Y11_15111* | Metallo-beta-lactamase family protein | | -1.74 |
| *Y11_01341* | Cytochrome c-type biogenesis protein CcmD,interacts with CcmCE | | -1.75 |
| *Y11_15021* | Lysr-family transcriptional regulatory protein | | -1.75 |
| *Y11_29561* | Ribose ABC transport system, ATP-binding protein RbsA | | -1.75 |
| *Y11_01721* | Transcriptional regulator, GntR family domain Aspartate aminotransferase (EC 2.6.1.1) | | -1.75 |
| *Y11_38591* | Protein ApaG | | -1.75 |
| *Y11_11041* | DNA-binding protein H-NS | | -1.76 |
| *Y11_33501* | Ketol-acid reductoisomerase (EC 1.1.1.86) (Acetohydroxy-acid isomeroreductase) (Alpha-keto-beta-hydroxylacil reductoisomerase) | | -1.78 |
| *Y11_07721* | Manganese ABC transporter, ATP-binding protein SitB | | -1.78 |
| *Y11_23671* | Uncharacterized protein | | -1.78 |
| *Y11_33421* | Acetolactate synthase small subunit (EC 2.2.1.6) | | -1.79 |
| *Y11_21361* | Yheo-like PAS domain | | -1.79 |
| *Y11_33941* | 2-haloalkanoic acid dehalogenase (EC 3.8.1.2) | | -1.82 |
| *Y11_39161* | DNA gyrase inhibitor YacG | | -1.83 |
| *Y11_21081* | UPF0345 protein Y11_21081 | | -1.84 |
| *Y11_03031* | Putative ABC transporter ATP-binding protein | | -1.84 |
| *Y11_06921* | Putative cytochrome | | -1.84 |
| *Y11_16671* | Uncharacterized protein | | -1.84 |
| *Y11_05421* | Putative transport protein | | -1.85 |
| *Y11_07981* | UDP-4-amino-4-deoxy-L-arabinose--oxoglutarate aminotransferase (EC 2.6.1.87) (UDP-(beta-L-threo-pentapyranosyl-4''-ulose diphosphate) aminotransferase) (UDP-4-amino-4-deoxy-L-arabinose aminotransferase) | | -1.86 |
| *Y11_11441* | Spovr-like protein | | -1.87 |
| *Y11_37931* | Uncharacterized protein | | -1.88 |
| *Y11_43231* | PTS system, glucitol/sorbitol-specific IIC component (EC 2.7.1.69) | | -1.88 |
| *Y11_13621* | Biotin synthesis protein bioC | | -1.89 |
| *Y11_43151* | Uncharacterized protein | | -1.90 |
| *Y11_15101* | Uncharacterized protein | | -1.91 |
| *Y11_14251* | Flagellar hook-length control protein FliK | | -1.91 |
| *Y11_20131* | Transcription repressor of multidrug efflux pump acrAB operon, TetR (AcrR) family | | -1.93 |
| *Y11_37461* | Acetyltransferase | | -1.94 |
| *Y11_17181* | Mgl repressor and galactose ultrainduction factor GalS, HTH-type transcriptional regulator | | -1.95 |
| *Y11_14901* | Glucose-1-phosphate adenylyltransferase (EC 2.7.7.27) (ADP-glucose pyrophosphorylase) (ADP-glucose synthase) | | -1.96 |
| *Y11_35721* | Hemin uptake protein HemP | | -1.96 |
| *Y11_39341* | Transcriptional regulator of AraC family,enterobactin-dependent, predicted | | -1.98 |
| *Y11_14811* | Beta-galactosidase (Beta-gal) (EC 3.2.1.23) (Lactase) | | -1.99 |
| *Y11_39631* | Ferric hydroxamate ABC transporter | | -2.00 |
| *Y11_27481* | Hipa protein | | -2.04 |
| *Y11_26141* | Uncharacterized protein | | -2.11 |
| *Y11_29521* | Ribose operon repressor | | -2.11 |
| *Y11_23901* | Conserved putative membrane protein | | -2.12 |
| *Y11_42571* | Uncharacterized protein | | -2.12 |
| *Y11_00591* | Sialic acid transporter (Permease) NanT | | -2.18 |
| *Y11_43411* | Putative exported protein | | -2.21 |
| *Y11_27471* | Uncharacterized protein | | -2.22 |
| *Y11_43241* | Probable lipid kinase YegS-like (EC 2.7.-.-) (EC 2.7.1.-) | | -2.25 |
| *Y11_12601* | Putative membrane protein YPO2012 | | -2.27 |
| *Y11_35741* | Putative heme iron utilization protein | | -2.28 |
| *Y11_06961* | Ferric reductase (1.6.99.14) | | -2.41 |
| *Y11_10431* | Transcriptional regulatory protein YciT | | -2.44 |
| *Y11_35761* | Threonine dehydratase, catabolic (EC 4.3.1.19) | | -2.45 |
| *Y11_13431* | Uncharacterized protein | | -2.47 |
| *Y11_05031* | Putative insecticidal toxin complex | | -2.47 |
| *Y11_28191* | Transcriptional (Co)regulator CytR | | -2.48 |
| *Y11_05441* | Bis(5'-nucleosyl)-tetraphosphatase (Asymmetrical) (EC 3.6.1.17) | | -2.54 |
| *Y11_04751* | Uncharacterized protein | | -2.59 |
| *Y11_15001* | Protein MtfA (Mlc titration factor A) | | -2.68 |
| *Y11_14701* | Universal stress protein C | | -2.70 |
| *Y11_08531* | Uncharacterized protein | | -2.79 |
| *Y11_35751* | Radical SAM family protein HutW, similar to coproporphyrinogen III oxidase, oxygen-independent,associated with heme uptake | | -2.91 |
| *Y11_31161* | Putative inner membrane protein | | -3.05 |
| *Y11_10441* | Uncharacterized protein | | -3.31 |
| *Y11_21251* | Transcriptional regulator, GntR family | | -3.66 |

**Table S5.** Bacterial genes differentially expressed between the late and the early time points of phiR1-37 infection. Log2FC value was calculated as the log_2_ of the ratio between the mean read counts of the negative control and phage infected samples.

| Name | Protein names | | Log2FC |
| --- | --- | --- | --- |
| *Y11_29791* | Phosphate ABC transporter, periplasmic phosphate-binding protein PstS | | 4.96 |
| *Y11_00861* | Putative exported protein | | 4.62 |
| *Y11_28711* | P pilus assembly/Cpx signaling pathway,periplasmic inhibitor | | 4.60 |
| *Y11_01281* | Uncharacterized protein | | 3.74 |
| *Y11_36501* | Putative exported protein YPO3518 | | 3.68 |
| *Y11_10461* | Osmotically inducible lipoprotein B | | 3.68 |
| *Y11_20021* | Uncharacterized protein | | 3.65 |
| *Y11_29801* | Phosphate transport system permease protein PstC | | 3.56 |
| *Y11_27571* | Putative inner membrane protein | | 3.47 |
| *Y11_05511* | Putative exported protein | | 3.37 |
| *Y11_08711* | Phage shock protein A | | 3.18 |
| *Y11_33481* | Rhs family protein | | 3.09 |
| *Y11_38001* | Osmotically inducible protein OsmY | | 3.09 |
| *Y11_38121* | Colicin type 7 (Cja) | | 2.99 |
| *Y11_23021* | Uncharacterized protein | | 2.94 |
| *Y11_21031* | Phosphate regulon transcriptional regulatory protein PhoB (SphR) | | 2.85 |
| *Y11_07931* | Uncharacterized protein | | 2.79 |
| *Y11_32171* | Uncharacterized protein | | 2.75 |
| *Y11_21161* | Putative exported protein YPO3518 | | 2.74 |
| *Y11_24451* | Transposase | | 2.67 |
| *Y11_20341* | Uncharacterized protein | | 2.61 |
| *Y11_19851* | Uncharacterized protein | | 2.57 |
| *Y11_04291* | Cold shock protein CspB | | 2.49 |
| *Y11_08721* | Phage shock protein B | | 2.47 |
| *Y11_04281* | Uncharacterized protein | | 2.43 |
| *Y11_07561* | Putative ABC sugar transporter | | 2.43 |
| *Y11_00801* | Putative exported protein | | 2.33 |
| *Y11_38811* | Tryptophanase (EC 4.1.99.1) | | 2.32 |
| *Y11_41911* | Putative exported protein | | 2.31 |
| *Y11_36811* | Serine transporter | | 2.31 |
| *Y11_29041* | Uncharacterized protein | | 2.29 |
| *Y11_38441* | Transcriptional activator NhaR | | 2.29 |
| *Y11_40311* | General secretion pathway protein C | | 2.29 |
| *Y11_09891* | Uncharacterized protein | | 2.28 |
| *Y11_38951* | Uncharacterized protein | | 2.27 |
| *Y11_12671* | Uncharacterized protein | | 2.24 |
| *Y11_40441* | Uncharacterized protein | | 2.24 |
| *Y11_17641* | Uncharacterized protein | | 2.23 |
| *Y11_42161* | Uncharacterized protein | | 2.21 |
| *Y11_24591* | Flp pilus assembly protein RcpC/CpaB | | 2.21 |
| *Y11_27291* | Cold shock protein CspG | | 2.21 |
| *Y11_20361* | Glycoprotein-polysaccharide metabolism | | 2.17 |
| *Y11_15071* | Exonuclease SbcC | | 2.17 |
| *Y11_25741* | Putative pertactin family virulence factor/autotransporter | | 2.17 |
| *Y11_04271* | Cold shock proteins | | 2.16 |
| *Y11_11501* | Acyl-[acyl-carrier-protein]--UDP-N-acetylglucosamine O-acyltransferase (EC 2.3.1.129) | | 2.16 |
| *Y11_34461* | sn-glycerol-3-phosphate transport system permease protein UgpA | | 2.15 |
| *Y11_08741* | Phage shock protein D | | 2.15 |
| *Y11_08731* | Phage shock protein C | | 2.14 |
| *Y11_18901* | Uncharacterized protein ybfE | | 2.12 |
| *Y11_22431* | Transcriptional regulator, ArsR family | | 2.12 |
| *Y11_33811* | Transposase | | 2.11 |
| *Y11_09741* | Uncharacterized protein | | 2.11 |
| *Y11_26961* | Uncharacterized protein | | 2.11 |
| *Y11_36261* | Upf0379 protein yjfY | | 2.10 |
| *Y11_10451* | L-ribulose-5-phosphate 4-epimerase (EC 5.1.3.4) | | 2.09 |
| *Y11_00231* | Uncharacterized protein | | 2.08 |
| *Y11_40791* | Amino-acid acetyltransferase (EC 2.3.1.-) (EC 2.3.1.1) (N-acetylglutamate synthase) | | 2.08 |
| *Y11_09621* | Uncharacterized protein | | 2.07 |
| *Y11_14731* | Mg(2+) transport ATPase protein C | | 2.06 |
| *Y11_38391* | Uncharacterized protein | | 2.05 |
| *Y11_06581* | Phage protein | | 2.05 |
| *Y11_19381* | Serine/threonine protein phosphatase (EC 3.1.3.16) | | 2.01 |
| *Y11_40361* | General secretion pathway protein F | | 2.01 |
| *Y11_26941* | Putative transcriptional regulator | | 2.00 |
| *Y11_31091* | Uncharacterized protein | | 1.99 |
| *Y11_19871* | Uncharacterized protein | | 1.98 |
| *Y11_07151* | Integral membrane protein | | 1.98 |
| *Y11_21221* | Flagellar brake protein YcgR (Cyclic di-GMP binding protein YcgR) | | 1.97 |
| *Y11_28671* | Replication gene B protein | | 1.94 |
| *Y11_00811* | Uncharacterized protein | | 1.93 |
| *Y11_24541* | Cobalt-zinc-cadmium resistance protein CzcA Cation efflux system protein CusA | | 1.93 |
| *Y11_19641* | Integrase |  | 1.92 |
| *Y11_31611* | Cob(I)alamin adenosyltransferase (EC 2.5.1.17) | | 1.92 |
| *Y11_37441* | Transposase | | 1.92 |
| *Y11_01291* | Putative transport | | 1.91 |
| *Y11_37731* | Phosphoenolpyruvate-dihydroxyacetone phosphotransferase, ADP-binding subunit DhaL (EC 2.7.1.121) | | 1.91 |
| *Y11_26921* | Protein AaeX | | 1.89 |
| *Y11_19631* | Inorganic pyrophosphatase/exopolyphosphatase | | 1.89 |
| *Y11_18771* | Cytolysin-activating lysine-acyltransferase rtxC | | 1.89 |
| *Y11_26301* | Uncharacterized protein | | 1.88 |
| *Y11_06011* | Cytolethal distending toxin subunit B | | 1.88 |
| *Y11_40331* | General secretion pathway protein D | | 1.88 |
| *Y11_20321* | Putative cytoplasmic protein | | 1.87 |
| *Y11_38731* | Putative membrane protein YPO4049 | | 1.86 |
| *Y11_30281* | Uncharacterized protein | | 1.86 |
| *Y11_06141* | Uncharacterized protein | | 1.86 |
| *Y11_30861* | PTS system, cellobiose-specific IIB component (EC 2.7.1.69) | | 1.86 |
| *Y11_02541* | Uncharacterized protein | | 1.85 |
| *Y11_05751* | Isocitrate dehydrogenase [NADP] (EC 1.1.1.42) | | 1.84 |
| *Y11_39221* | Type IV pilin PilA | | 1.83 |
| *Y11_23181* | 3-phenylpropionate dioxygenase ferredoxin subunit | | 1.83 |
| *Y11_01021* | Putative lipoprotein | | 1.83 |
| *Y11_32411* | Nitrite transporter NirC | | 1.81 |
| *Y11_23541* | Uncharacterized protein | | 1.81 |
| *Y11_21021* | Phosphate regulon sensor protein PhoR (SphS) (EC 2.7.13.3) | | 1.81 |
| *Y11_34291* | Uncharacterized protein | | 1.81 |
| *Y11_29971* | Methionine ABC transporter ATP-binding protein | | 1.79 |
| *Y11_40431* | General secretion pathway protein L | | 1.78 |
| *Y11_05001* | Multidrug-efflux transporter, major facilitator superfamily (MFS) | | 1.77 |
| *Y11_24621* | Type II/IV secretion system ATPase TadZ/CpaE,associated with Flp pilus assembly | | 1.77 |
| *Y11_01271* | Alkylphosphonate utilization operon protein PhnA | | 1.75 |
| *Y11_30611* | Xylose ABC transporter, periplasmic xylose-binding protein XylF | | 1.73 |
| *Y11_28241* | Regulator of ribonuclease activity A | | 1.71 |
| *Y11_15771* | Protein ImpG/VasA | | 1.70 |
| *Y11_37721* | Phosphoenolpyruvate-dihydroxyacetone phosphotransferase, dihydroxyacetone binding subunit DhaK (EC 2.7.1.121) | | 1.69 |
| *Y11_02831* | Putative cI prophage repressor protein | | 1.69 |
| *Y11_24481* | HTH-type transcriptional regulator gadW | | 1.69 |
| *Y11_38741* | Putative membrane protein | | 1.67 |
| *Y11_29811* | Phosphate transport system permease protein PstA | | 1.67 |
| *Y11_17761* | Fatty acid desaturase | | 1.67 |
| *Y11_25941* | Inner membrane protein YqjK | | 1.67 |
| *Y11_12181* | ISPsy11, transposase OrfA | | 1.67 |
| *Y11_17221* | Putative MFS Superfamily transporter | | 1.66 |
| *Y11_07941* | Putative exported protein | | 1.66 |
| *Y11_22461* | Transcriptional regulator, MerR family | | 1.65 |
| *Y11_05161* | Putative cytoplasmic protein | | 1.64 |
| *Y11_00681* | Macrolide-specific efflux protein MacA | | 1.63 |
| *Y11_37741* | Phosphoenolpyruvate-dihydroxyacetone phosphotransferase, subunit DhaM DHA-specific IIA component DHA-specific phosphocarrier protein HPr DHA-specific EI component (EC 2.7.1.121) | | 1.63 |
| *Y11_14321* | Flagellar biosynthetic protein fliR | | 1.63 |
| *Y11_34281* | RNA polymerase sigma factor | | 1.63 |
| *Y11_33531* | Colicin immunity protein | | 1.62 |
| *Y11_35941* | Quaternary ammonium compound-resistance protein sugE | | 1.61 |
| *Y11_02801* | Putative methyl-accepting chemotaxis protein | | 1.60 |
| *Y11_39371* | UPF0231 protein Y11_39371 | | 1.59 |
| *Y11_19421* | Uncharacterized protein | | 1.58 |
| *Y11_09661* | Uncharacterized protein | | 1.58 |
| *Y11_38431* | Na(+)/H(+) antiporter NhaA (Sodium/proton antiporter NhaA) | | 1.58 |
| *Y11_25931* | Inner membrane protein YqjE | | 1.58 |
| *Y11_33161* | Uncharacterized protein | | 1.57 |
| *Y11_10821* | Outer membrane protein W | | 1.57 |
| *Y11_09811* | UPF0482 protein Y11_09811 | | 1.55 |
| *Y11_36891* | Ornithine decarboxylase (EC 4.1.1.17) | | 1.55 |
| *Y11_38291* | Uncharacterized protein | | 1.55 |
| *Y11_09371* | Putative inner-membrane regulatory protein | | 1.54 |
| *Y11_33151* | HTH-type transcriptional regulator zntR | | 1.54 |
| *Y11_41631* | Putative lipoprotein signal peptide | | 1.54 |
| *Y11_12421* | Putative inner membrane protein | | 1.54 |
| *Y11_02281* | Uncharacterized protein | | 1.53 |
| *Y11_42191* | Autonomous glycyl radical cofactor | | 1.53 |
| *Y11_17651* | Outer membrane protein C | | 1.53 |
| *Y11_31781* | Multiple antibiotic resistance protein marC | | 1.53 |
| *Y11_23171* | Putative metalloprotease yggG | | 1.51 |
| *Y11_10701* | Uncharacterized protein | | 1.50 |
| *Y11_31461* | Universal stress protein B | | 1.50 |
| *Y11_23721* | Ferric anguibactin transport system permease protein fatD | | -1.51 |
| *Y11_33941* | 2-haloalkanoic acid dehalogenase (EC 3.8.1.2) | | -1.54 |
| *Y11_23701* | Petrobactin ABC transporter, permease protein II | | -1.55 |
| *Y11_29561* | Ribose ABC transport system, ATP-binding protein RbsA | | -2.04 |

**A**


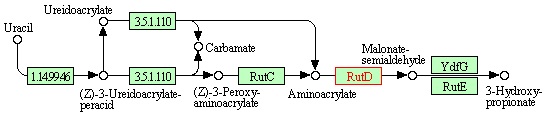


**B**


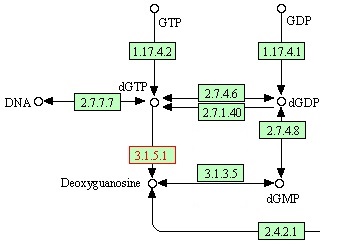


**C**

| Gene ID | Protein | Log2FC | |
| --- | --- | --- | --- |
|  |  | Early response | Late response |
| Y11_39741 | Deoxyguanosinetriphosphate triphosphohydrolase (dGTP triphosphohydrolase) (dGTPase) (EC 3.1.5.1) | -1.99 | -1.73 |
| Y11_13711 | Putative aminoacrylate hydrolase RutD (EC 3.5.1.-) (Aminohydrolase) | -1.56 | -0.43 |

**Figure S6.** The nucleotide metabolism. The parts of bacterial purine (A) and pyrimidine (B) metabolic pathways affected by φR1-37 infection. The genes differentially expresses upon the phage infection are marked with red color. The graph was prepared based on the KEGG Metabolic Pathways (<http://www.genome.jp/kegg/kegg2.html>). The log2FC values obtained for the early and late response for the genes marked in the graphs are mentioned below (C).
